# Twin-Engine Janus Supramolecular Nanomotors with Counterbalanced Motion

**DOI:** 10.1021/jacs.2c02682

**Published:** 2022-06-14

**Authors:** Jingxin Shao, Shoupeng Cao, Hailong Che, Maria Teresa De Martino, Hanglong Wu, Loai K. E. A. Abdelmohsen, Jan C. M. van Hest

**Affiliations:** Bio-Organic Chemistry, Institute for Complex Molecular Systems, Eindhoven University of Technology, P.O. Box 513 (STO 3.41), 5600 MB Eindhoven, The Netherlands

## Abstract

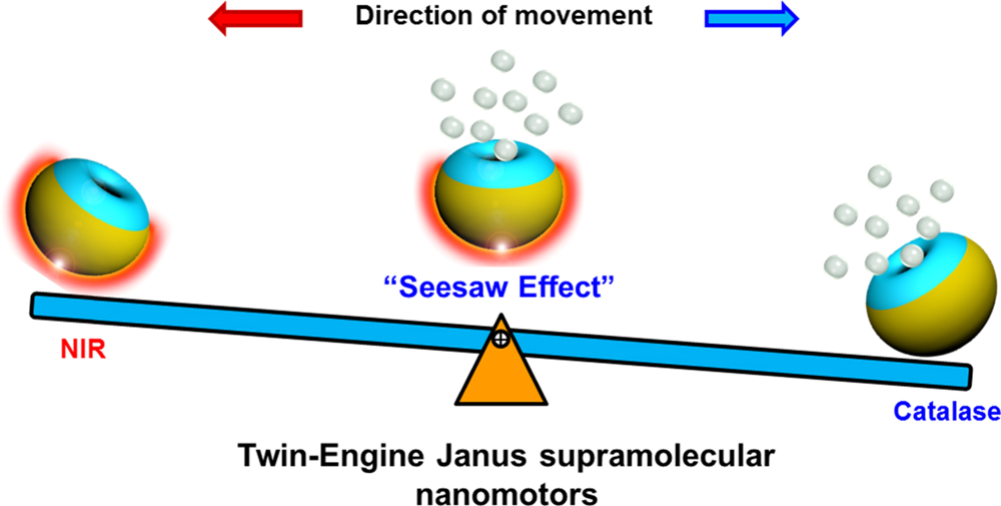

Supramolecular nanomotors were created with two types of propelling forces that were able to counterbalance each other. The particles were based on bowl-shaped polymer vesicles, or stomatocytes, assembled from the amphiphilic block copolymer poly(ethylene glycol)-*block*-polystyrene. The first method of propulsion was installed by loading the nanocavity of the stomatocytes with the enzyme catalase, which enabled the decomposition of hydrogen peroxide into water and oxygen, leading to a chemically induced motion. The second method of propulsion was attained by applying a hemispherical gold coating on the stomatocytes, on the opposite side of the opening, making the particles susceptible to near-infrared laser light. By exposing these Janus-type twin engine nanomotors to both hydrogen peroxide (H_2_O_2_) and near-infrared light, two competing driving forces were synchronously generated, resulting in a counterbalanced, “seesaw effect” motion. By precisely manipulating the incident laser power and concentration of H_2_O_2_, the supramolecular nanomotors could be halted in a standby mode. Furthermore, the fact that these Janus stomatocytes were equipped with opposing motile forces also provided a proof of the direction of motion of the enzyme-activated stomatocytes. Finally, the modulation of the “seesaw effect”, by tuning the net outcome of the two coexisting driving forces, was used to attain switchable control of the motile behavior of the twin-engine nanomotors. Supramolecular nanomotors that can be steered by two orthogonal propulsion mechanisms hold considerable potential for being used in complex tasks, including active transportation and environmental remediation.

Nanoscopic particles with motile features have become a topic of intensive investigation over the past years. Inspired by natural motor systems, a range of different particles with a variety of propulsion mechanisms have been developed.^1−5^ They can convert local energy from their surroundings into locomotion in a fluidic environment, which can be utilized for the completion of complex tasks.^6−10^ Customized autonomous nanomotors have been created for sensing,^11,12^ environmental remediation,^13,14^ energy (hydrogen–oxygen fuel cell),^15^ and biomedical applications.^16−20^ The majority of first-generation nanomotors was propelled by catalytic decomposition of chemical fuels, such as the bubble-pair propelled colloidal kayakers.^21^ However, in fuel-deprived environments, the motion of this kind of nanomotor is inevitably suppressed. To tackle this issue, fuel-free motors that can be powered and remotely guided by external physical stimuli (e.g., magnetic fields, ultrasound, and light) have been successfully constructed.^22−25^ Most of the current artificial nanomotors are however still powered by a single engine, which is accompanied by some limitations.

A major shortcoming of a single-mode nanomotor is the difficulty in precisely manipulating and modulating the motile behavior on-demand.^26,27^ To address this challenge, a new generation of nanomotors with multimode propulsion thus needs to be further developed. Considerable efforts have been devoted to realizing this class of artificial motors.^28,29^ To date, several dual-driven micro/nanomotors based on hybrid materials have been created, which are propelled by chemical–ultrasound,^30^ chemical–magnetic,^31^ chemical–light,^32,33^ ultrasound–magnetic,^34^ and ultrasound–light energy sources.^35^ Compared to the single-engine micro/nanomotors, incorporation of two propulsion mechanisms in one motor makes them more robust in complex surroundings and less affected by the constraints on the availability of fuel resources or other environmental parameters. Importantly, besides addressing the limitations of traditional single-mode motors, two propulsion modes working together enable a better control over the directionality and precision of motion, thereby expanding the application potential of micro/nanomotor systems. However, among these dual-driven micro/nanomotors, only a few were reported to have a controllable stalling of motion through the “seesaw effect”, where opposing motile forces counterbalance each other.

To allow directed motion, asymmetry should be installed in the particle design. A very effective approach is the construction of Janus polymeric particles/capsules with a hemispherical platinum or gold shell; upon exposure to hydrogen peroxide (H_2_O_2_) or near-infrared (NIR) laser irradiation, respectively, autonomous motion is introduced.^36−39^ Another useful chassis for nanomotor design was recently developed by our group and is based on stomatocytes. Stomatocytes are polymeric vesicles with a unique bowl-shaped morphology, created via an osmotic-induced shape change process of spherical vesicles composed of amphiphilic block copolymers.^40−43^ By loading the well-defined cavity with catalytic nanoparticles or enzymes, stomatocyte-based catalytic nanomotors were created.^44,45^ Even enzymatic networks could be effectively incorporated for this purpose.^46^ The introduction of Janus morphology on an enzyme-filled stomatocyte would allow the construction of an efficient dual-powered supramolecular nanomotor.

Herein, we present a twin-engine supramolecular nanomotor, which is based on stomatocytes assembled from the amphiphilic block copolymer poly(ethylene glycol)-block-polystyrene (PEG-*b*-PS). The responsiveness to chemical fuel was included by loading the stomatocyte nanocavity with the enzyme catalase during the process of shape transformation. For the installment of a second driving force, a hemispherical gold layer was introduced onto the stomatocytes by sputter coating, on the other side of the opening, which made the particles susceptible to NIR light. The dual-functional nanomotors displayed efficient propulsion upon either irradiation with NIR light (NIR driven mode) or in the presence of H_2_O_2_ (enzyme driven mode). A “seesaw effect” was observed when both forces were applied simultaneously, since the NIR driving force generated on the gold side counterbalanced the enzymatic propulsion force. Because of the observed “seesaw effect”, we experimentally confirmed the movement direction of the enzyme-propelled stomatocytes with the cavity pointing away from the direction of motion. More importantly, a high level of control over motion was achieved by tuning the net outcome of the two coexisting driving forces via regulating the incident laser power.

To investigate the twin-engine nanomotor features, three different supramolecular nanomotors were constructed: stomatocytes coated with a hemispherical gold layer (motor 1: Janus stomatocytes), which could be propelled solely by NIR light; catalase-filled stomatocytes (motor 2: catalase stomatocytes), which responded to H_2_O_2_, and the combined system in which both functionalities were included. Their method of preparation is schematically depicted in Figure 1. All stomatocytes were assembled from the amphiphilic block copolymer poly(ethylene glycol)-polystyrene (PEG_45_-*b*-PS_230_), which was synthesized by using atom-transfer radical polymerization as reported in previously published work (Figure S1).^47^

**Figure 1:**
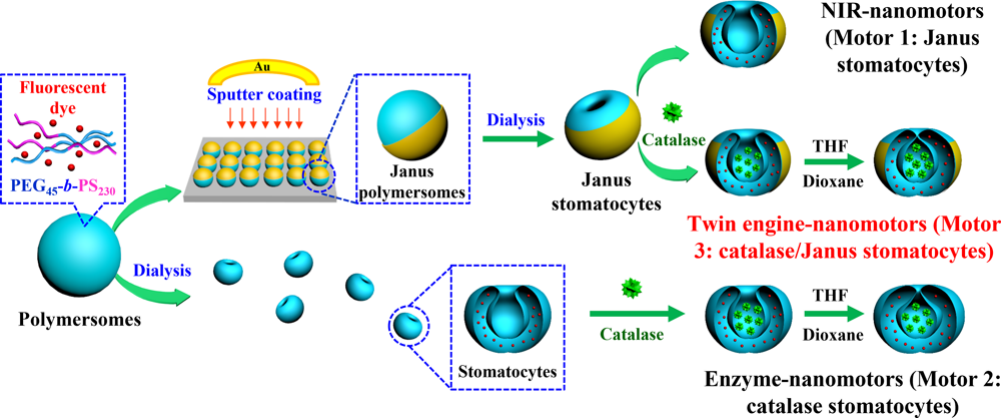
Schematic depiction of the designing and supramolecular assembly of the stomatocyte-based nanomotors, including NIR-driven Janus nanomotors (motor 1: Janus stomatocytes), enzyme-driven nanomotors (motor 2: catalase stomatocytes), and twin-engine Janus nanomotors (motor 3: catalase/Janus stomatocytes).

The formation of the bowl-shaped stomatocytes was conducted according to established protocols. In short, after assembling the block copolymer into spherical vesicles, by addition of water to a polymer solution in organic solvent, dialysis was conducted to initiate an osmotic shock-induced shape change process.^43^ The morphology of the stomatocytes was confirmed by scanning electron microscopy (SEM), as shown in Figure S1C. To prepare the Janus stomatocytes, the above-mentioned method was modified. Before the shape change process was employed, the spherical polymersomes were deposited on a silica wafer into a monolayer via drop-casting. Subsequently, this was followed by sputter coating of a gold (Au) layer on top of the polymersomes. The Janus polymersomes were released from the substrate into solution via ultrasound treatment. Next, they were transferred to a dialysis bag to induce the shape transformation (Figure 2A). Due to the presence of the hemispherical gold layer, only the uncoated polymer side showed sufficient flexibility to undergo a shape transformation, which resulted in indention of the vesicles mainly on the opposite side of the gold layer. This specific morphology was observed by SEM and transmission electron microscopy (TEM) (Figures 2B–D and S2). Energy-dispersive X-ray (EDX) mapping of Au on a number of Janus stomatocytes further confirmed the existence of a hemispherical Au layer on the “bowl bottom” part of the stomatocyte, away from the opening (Figure 2E,F). Dynamic light scattering (DLS) data furthermore indicated that the average hydrodynamic size of Janus stomatocytes was almost comparable to those of native stomatocytes, 435.3 and 410.7 nm with polydispersity index of 0.107 and 0.027, respectively (Figure S3).

**Figure 2:**
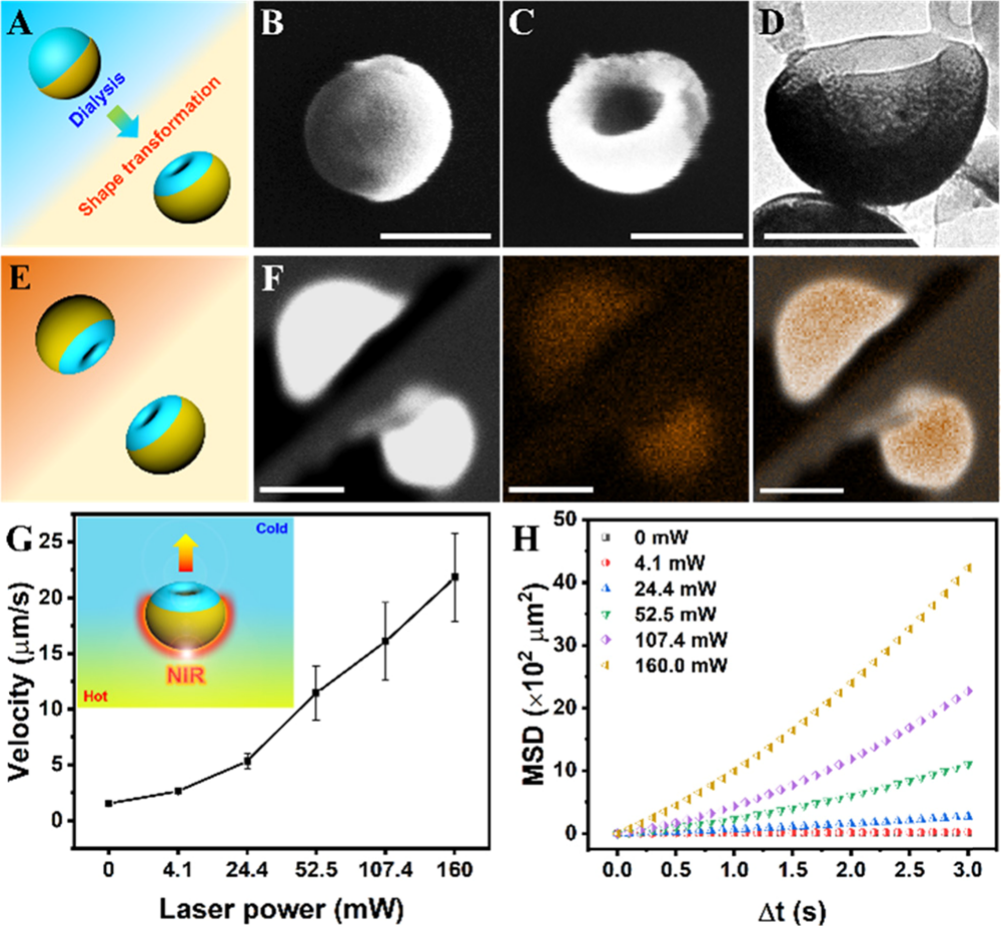
Preparation and characterization of Janus stomatocyte-based supramolecular nanomotors (motor 1: Janus stomatocytes) and their NIR triggered motion. (A) Schematic illustration of the construction of Janus stomatocytes via dialysis treatment. (B) SEM image of a Janus polymersome before dialysis, scale bar = 200 nm. (C) SEM image of a Janus stomatocyte after dialysis, scale bar = 200 nm. (D) TEM image of a Janus stomatocyte, scale bar = 200 nm. (E) Schematic depiction of the orientation of Janus stomatocytes for energy-dispersive X-ray spectroscopy (EDX) elemental mapping analysis. (F) Elemental mapping of Janus stomatocytes by EDX showing the Janus morphology of stomatocytes. From left to right: electron image, EDX mapping image of Au, and merged image. Scale bar = 500 nm. (G) Velocity dependence of the Janus stomatocytes on the NIR output laser power. (H) Mean square displacement (MSD) of Janus stomatocytes versus time interval (Δ*t*) analyzed from motion tracking trajectories.

By coating stomatocytes with a hemispherical Au coating, Janus stomatocytes were obtained, which were photoactivatable via the surface plasmon resonance features of the Au layer. According to established theories, thermophoresis is thereby the main propulsion mechanism.^48−51^ Under NIR irradiation, the temperature of the surrounding medium around a Janus particle is spatially nonuniform, resulting in inhomogeneous thermal fluctuations, which leads to particle motion. To investigate the movement behavior, two photon-confocal laser scanning microscopy (TP-CLSM) was used to observe and record the laser power-dependent motion. The trajectory of randomly selected Janus stomatocytes (the number of nanomotors *n* = 20) was tracked from the recorded video by ImageJ (Figure S4). The motion of Janus stomatocytes was indeed laser power dependent, since with the increase of output laser power, a higher velocity was achieved (Figure 2G). The mean squared displacement (MSD) as a function of NIR laser power was calculated according to previously published methods,^52,53^ which further confirmed that the motion of Janus stomatocytes is strongly reliant on the incident laser power (Figure 2H).

As a second control, enzyme-filled stomatocytes (motor 2: catalase stomatocytes) were prepared according to earlier published protocols. Catalase was used, as it is highly efficient in decomposing H_2_O_2_ into water and oxygen, and has often shown its value in the research of active particles.^54,55^ As catalase is an enzyme very sensitive to organic solvents, we adopted a mild methodology, developed in our group, to load catalase into the nanocavity of stomatocytes.^44,45^ Briefly, spherical polymersomes were prepared by the solvent exchange method, followed by dialysis-induced shape transformation to stomatocytes. This process was quenched in an early stage by the addition of an excess of water to attain stomatocytes with a wide-open neck. Then, the shape change process was continued in the presence of catalase and a small fraction of organic solvent to resolubilize the membrane. After the shape change process, the opening of the stomatocytes was significantly diminished to prevent enzyme leakage. Morphological and size changes were followed by SEM and DLS, respectively (Figure S5). Asymmetric flow field flow fractionation (AF4) coupled to multiangle light scattering and DLS were used to confirm the encapsulation of the enzyme (Figure S6). The motility of the catalase stomatocytes as a function of H_2_O_2_ concentration was determined by TP-CLSM. Figure S7A displays tracks of randomly selected enzyme nanomotors in the presence of H_2_O_2_. Based on the trajectories, an average velocity and MSD of catalase stomatocytes were calculated (Figures S7B and S7C). These data demonstrate that the motion of catalase stomatocytes was H_2_O_2_ concentration dependent, since more directional motion and higher speeds were achieved when increasing the H_2_O_2_ concentration.

Next, the two propulsion mechanisms were combined in the same particle, yielding twin-engine Janus supramolecular nanomotors (motor 3: catalase/Janus stomatocytes, Figure 3A). We first created Au-coated Janus polymersomes as described for the preparation of motor 1; these were shape changed into wide-necked Janus stomatocytes. Then, the same protocol for entrapment of catalase into stomatocytes was conducted, as described for motor 2. SEM measurements confirmed the overall shape transformation from spherical Janus polymersomes and wide-open Janus stomatocytes to narrow neck Janus stomatocytes (Figures 3B,C and S8). To analyze the efficiency of catalase encapsulation, we compared the enzyme loading and activity between stomatocytes and Janus stomatocytes using a BCA protein assay and catalase activity assay (Figures S9 and S10). From these data, we experimentally verified that both types of stomatocytes showed similar enzyme features. The size of the Janus stomatocytes was slightly increased during the process possibly because of the presence of the hemispherical Au coating.

**Figure 3:**
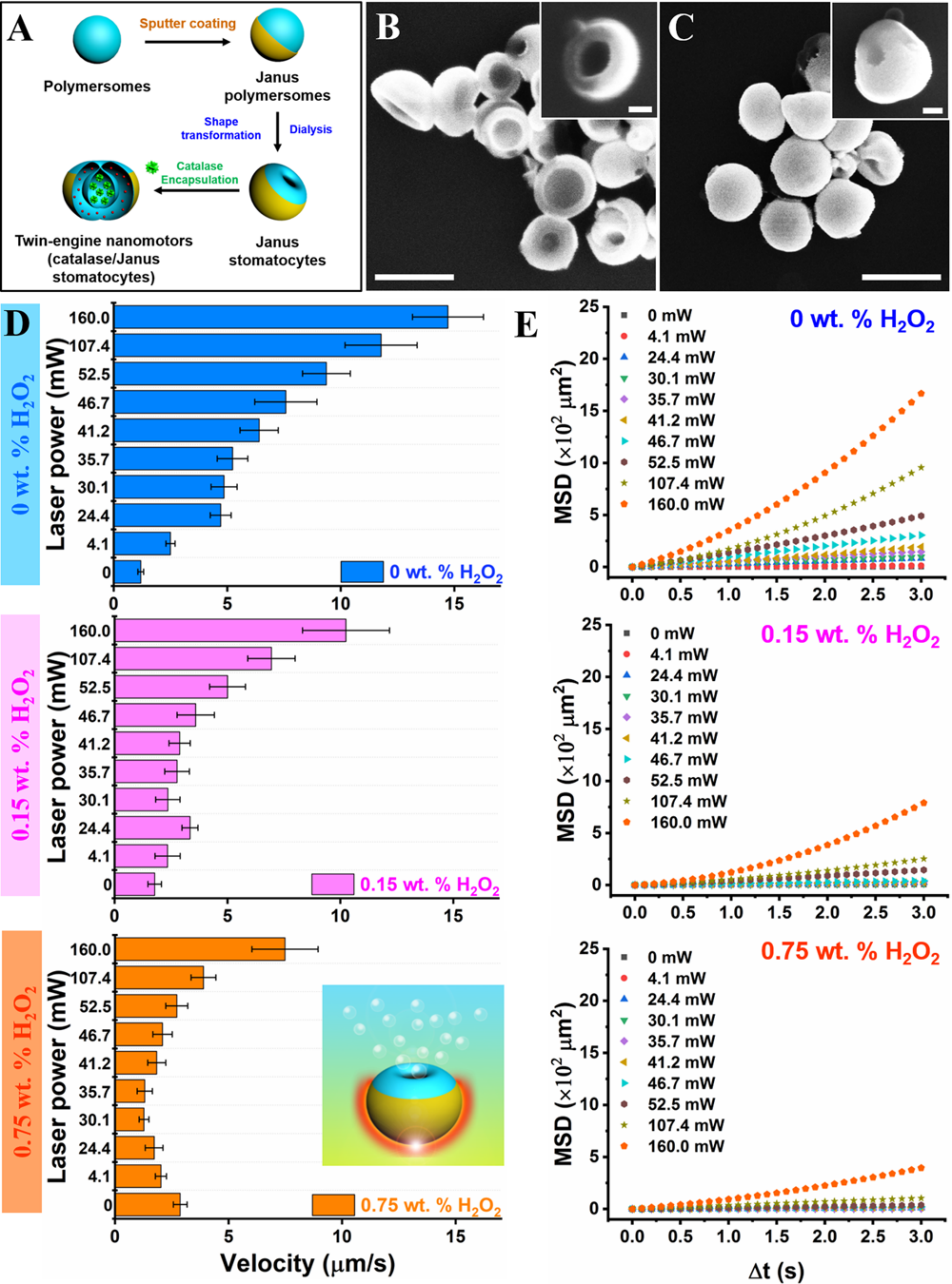
Movement analysis of twin-engine Janus stomatocyte-based nanomotors (motor 3: catalase/Janus stomatocytes). (A) Schematic representation of the steps involved in the preparation of twin-engine catalase/Janus stomatocytes. (B,C) SEM images of Janus stomatocytes (left) and catalase/Janus stomatocytes (right). Scale bar = 500 nm (inset = 100 nm). (D) Velocity of catalase/Janus stomatocytes at different laser powers in the presence of 0 wt % H_2_O_2_ (blue), 0.15 wt % H_2_O_2_ (pink), and 0.75 wt % H_2_O_2_ (orange). (E) MSDs of twin-engine catalase/Janus stomatocytes irradiated with different laser powers in the presence of 0, 0.15, and 0.75 wt % H_2_O_2_.

First, the propulsion performance of the catalase/Janus stomatocytes under single-mode conditions was investigated using TP-CLSM (Figures 3D,E and S11). To enhance the traceability of the catalase/Janus stomatocytes, doxorubicin (Dox) was used as a fluorescent dye, which was loaded during the formation of polymersomes. Under NIR irradiation, catalase/Janus stomatocytes were photoactivated and exhibited the expected behavior, namely, an increase in velocity, MSD, and moving distance with enhanced laser power. Next, we investigated the enzyme-activated motion by the addition of H_2_O_2_ fuel at different concentrations (0.15 wt % H_2_O_2_ and 0.75 wt % H_2_O_2_). The directed motion of the enzyme nanomotors was strongly dependent on the fuel concentration. Upon increasing the concentration of H_2_O_2_, particle velocity and MSD were enhanced.

Next, the nanomotors were exposed to both driving forces. To simplify the analysis process, we investigated the movement behavior of catalase/Janus stomatocytes as a function of NIR laser power in the presence of a fixed concentration of H_2_O_2_ fuel. When the experiment was performed in the presence of 0.15 wt % H_2_O_2_, particle velocity increased with the increasing NIR output laser power. However, when the same experiment was performed at 0.75 wt % H_2_O_2_, the velocity first decreased, before an increase could be observed at a higher NIR laser power (Figure 3D,E), leading to a minimum particle velocity at a specific fuel concentration and NIR laser power. This behavior is explained by the fact that the driving forces generated by catalytic decomposition of H_2_O_2_ and the NIR-induced photothermal effect oppose each other, resulting in a “seesaw effect” of the supramolecular nanomotors (Figure S12A). As both the enzyme-driven system and the photothermal effect have to be directional to counterbalance each other, this experiment also provides direct proof of the motion direction of enzyme-propelled stomatocytes. The photothermal effect creates a temperature gradient around the gold shell, which drives the particles away from the source of heating; the particles move with the stomatocyte cavity to the front and the Au layer to the back. This motion can only be compensated if the enzyme-driven propulsion does exactly the opposite. This means that the cavity is pointing to the rear, from which oxygen or oxygen bubbles can escape to install directed motion in the stomatocytes.

Having established the conditions under which the two forces were in balance, we could create a “stop-and-go” situation by switching the laser power on and off, respectively (Figures 4 and S12). This method of control was easier to achieve than to tune the H_2_O_2_ fuel concentration.

**Figure 4:**
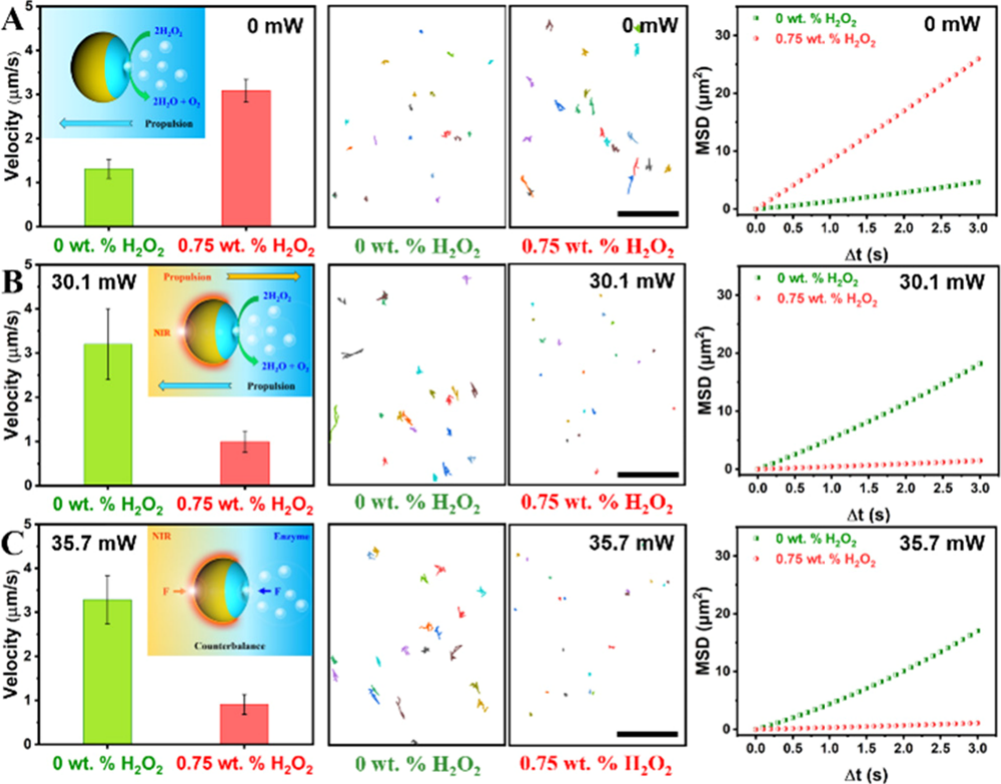
“Seesaw effect” of twin-engine Janus supramolecular nanomotors (catalase/Janus stomatocytes) via precisely controlling the motion. Velocity, tracking trajectories, and MSD of catalase/Janus stomatocytes in the presence of 0 wt % H_2_O_2_ or 0.75 wt % H_2_O_2_, and irradiation with (A) 0 mW, (B) 30.1 mW, and (C) 35.7 mW NIR laser light. All scale bars in the tracking trajectories correspond to 50 μm. Inserted schematic figures display the motion behavior of catalase/Janus stomatocytes in response to the different conditions. In the absence of NIR (inset image in A), catalase/Janus stomatocytes exhibit the properties of regular enzyme-powered nanomotors, which move faster by increasing the concentration of hydrogen peroxide fuel. Upon NIR illumination (inset image in B), the photothermal effect around catalase/Janus stomatocytes results in motion in the opposite direction of the motion induced by the enzyme-driven pathway. Two opposing forces generated on catalase/Janus stomatocytes are counterbalanced under specific conditions, resulting in halting the motion of the twin-engine Janus motors (inset image in C).

Based on the studies reported in Figure 3, the nanomotors were exposed to three different output laser powers, 0, 30.1, and 35.7 mW, as the latter two should allow minimal motion when applied in the presence of 0.75 wt % H_2_O_2_. As shown in Figure 4A, catalase/Janus stomatocytes displayed regular fuel concentration-dependent motion behavior in the absence of NIR laser irradiation. The average speed of catalase/Janus stomatocytes was increased from 1.31 to 3.09 μm/s with the increasing H_2_O_2_ concentration (Supporting Information, Videos S1 and S2). An apparent decrease in velocity was observed after switching the NIR laser on (Figure 4B,C). Movement trajectories of catalase/Janus stomatocytes extracted from recorded videos (Supporting Information, Videos S3–S5) displayed that the nanomotors indeed entered a “static state” by exposing them both to 0.75 wt % H_2_O_2_ fuel and NIR laser light of certain laser power (30.1 and 35.7 mW). Inset figures in Figure 4 depict the mechanism of the “seesaw effect” induced by opposing forces. This method provides excellent control over the motion of the particles.

To further demonstrate the ability to control stomatocytes’ motion using light and chemical fuel, nanoparticle tracking analysis equipped with an external laser source was utilized, as shown in Figure 5A. Here, we aimed at studying the ability of the stomatocytes to be activated and deactivated using light in the presence of H_2_O_2_. Indeed, MSD analysis and motion trajectories of the nanomotors showed alteration of motion between non-Brownian and Brownian upon switching the light on and off, respectively. Three different driven modes existed; these included two single modes (i.e., laser- and enzyme-driven modes), as well as a dual mode (i.e., two driving forces coexisting in one nanomotor). In the absence of H_2_O_2_, the catalase/Janus stomatocytes were propelled in single mode (laser-driven mode), resulting in laser power-dependent motion (Figure 5B, Supporting Information, Video S6). In the presence of H_2_O_2_ and absence of light, the motion of catalase/Janus stomatocytes exhibited the enzyme-driven mode, as presented in Figure 5C. Precise motion control was achieved by switching between single and dual driven modes, as demonstrated by velocity, MSD, and motion trajectories of the nanomotors (Figures 5D and S13, Supporting Information, Video S7). It is worth mentioning that the directional motion of such a stomatocyte platform is in line with previous reports.^56^ Additionally, the importance of the gold layer for light-mediated motion was confirmed by investigating the behavior of uncoated stomatocytes (without gold) upon laser irradiation (Figure S14). MSD and trajectory analysis did not show any difference in motion of such uncoated stomatocytes upon light irradiation.

**Figure 5:**
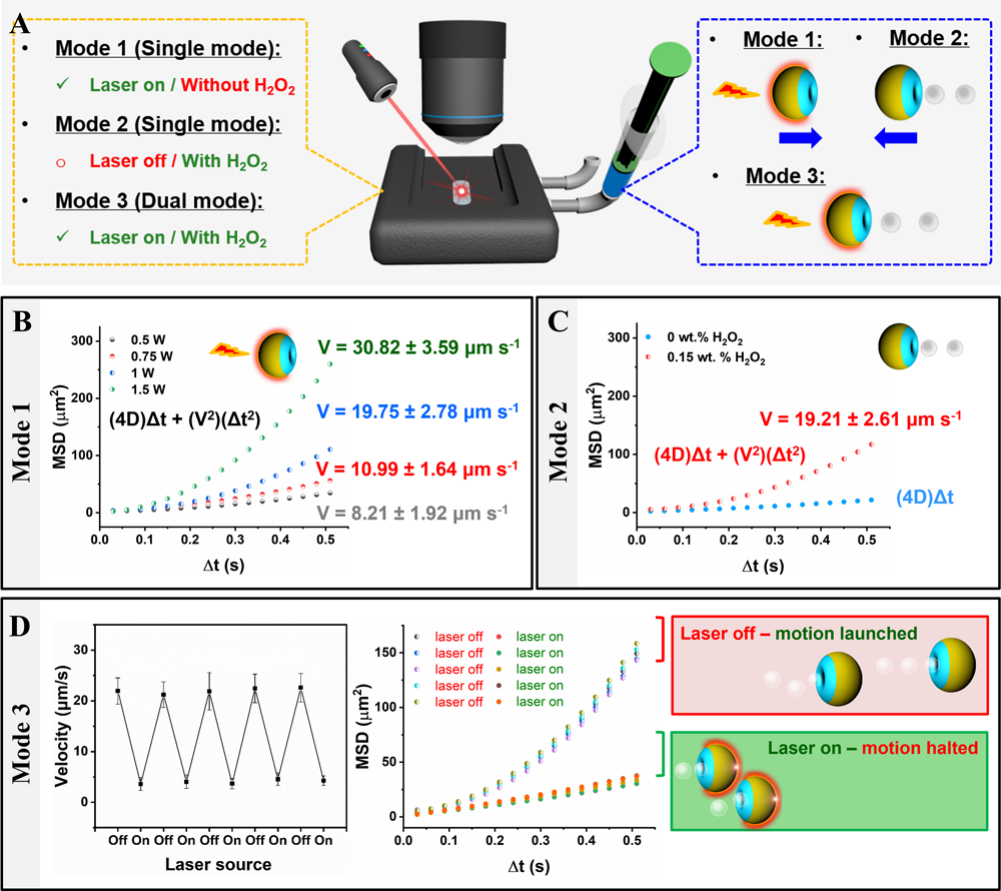
Programmable motion of catalase/Janus stomatocytes. (A) Schematic depicting the characterization of motion behavior with single (Mode 1 and Mode 2) or dual mode (Mode 3) by nanoparticle tracking analysis (NTA). (B) Motion characterization of catalase/Janus stomatocytes propelled by laser irradiation via MSD calculation (Mode 1). The velocities were calculated theoretically from (4D)Δ*t* + (*V*^2^)(Δ*t*^2^). (C) MSD of catalase/Janus stomatocytes in the presence of H_2_O_2_ without laser irradiation (Mode 2). (D) Velocity of dual mode propelled motion (Mode 3) of catalase/Janus stomatocytes (1 W, 0.15 wt % H_2_O_2_) (left) and MSD of catalase/Janus stomatocytes (right). Switchable motion between dual and single mode was manipulated by tuning laser input.

Finally, we investigated the collective behavior of the catalase/Janus stomatocytes. Pioneering work demonstrated that self-propelled particles prefer to accumulate in a region of space and have a strong tendency to form clusters, compared to passive particles.^57−62^ For instance, light-propelled micromotors based on the photothermal effect formed cluster structures, due to light-induced convection.^63−65^ Convection is generated in the liquid surrounding the particles, due to the temperature gradient between the NIR-irradiated region and unexposed region.^66^ As our catalase/Janus stomatocytes are also driven by a photothermal effect, they should also be susceptible to a swarming behavior. However, when the particles are simultaneously operated by light and chemical fuel, the “seesaw effect” could influence the swarming behavior of the catalase/Janus stomatocytes. Different motion states of the catalase/Janus stomatocytes might be obtained, according to the net outcome of the oppositely propelling forces. To explore these states, we used TP-CLSM to record the collective motion of the catalase/Janus stomatocytes under single and dual propulsion modes. As shown in Figures S15A to S15B, the catalase/Janus stomatocytes powered by both forces (laser on/with H_2_O_2_) exhibited a halted state, due to the counterbalance between the two coexisting forces. As a result, the fluorescence intensity of the time lapsed CLSM images analysis by ImageJ did not change as there was no cluster formation in the “static state”. In contrast, the dynamic state was achieved once switched to single mode, only applying the photothermal effect (laser on/no H_2_O_2_). The catalase/Janus stomatocytes tended to form clusters during the observed periods, resulting in an increase in fluorescence intensity observed in the time lapsed CLSM images (Figures S15C to S15D).

In summary, we have designed a twin-engine supramolecular nanomotor based on bowl-shaped polymer vesicles or stomatocytes. Encapsulation of the enzyme catalase in the nanocavity of the stomatocytes provided the particles with the ability to undergo motion induced by the biocatalytic conversion of H_2_O_2_. By decorating the stomatocytes with a hemispherical gold shell opposite of the opening of the nanocavity, the particles were susceptible to NIR laser light and, consequently, were propelled via the photothermal effect. We demonstrated that the two modes of motion operate in different directions, which allowed us to create a “seesaw effect” with these supramolecular nanomotors; by tuning both the fuel concentration and incident laser power, particles were severely slowed down through balancing the two competitive propulsion modes. Moreover, the swarming behavior of the catalase/Janus stomatocytes could be tailored by adjusting the propulsion modes, which further demonstrated the versatility in motion control of these dual engine nanomotors, which could not be obtained by using only one propulsion mode. Given their attractive performance, the new twin-engine supramolecular nanomotors are expected to broaden the practical applications of nanomotors, ranging from on-demand assembly, environmental analysis, and sensing to activities in the biomedical field.

## Supporting Information Available

Materials, methods, and characterization of block copolymers, stomatocytes, Janus stomatocytes, catalase encapsulation, and movement behaviors (PDF)
(Supporting Movie S1) Motion video of catalase/Janus stomatocytes in the absence of H_2_O_2_ fuel and the NIR laser; (Supporting Movie S2) motion video of catalase/Janus stomatocytes in a 0.75 wt % H_2_O_2_ solution without NIR laser irradiation; (Supporting Movie S3) motion of catalase/Janus stomatocytes in a 0 wt % H_2_O_2_ solution under NIR laser irradiation (30.1 mW); (Supporting Movie S4) twin-engine mode of catalase/Janus stomatocytes (0.75 wt % H_2_O_2_ solution/30.1 mW NIR laser); (Supporting Movie S5) twin-engine mode of catalase/Janus stomatocytes (0.75 wt % H_2_O_2_ solution/35.7 mW); (Supporting Movie S6) motion video of catalase/Janus stomatocytes upon laser irradiation (1 W); and (Supporting Movie S7) motion video of catalase/Janus stomatocytes in a 0.15 wt % H_2_O_2_ solution under laser irradiation (1 W) (ZIP)

## References

[ref1] Palagi S.; Fischer P. Bioinspired Microrobots. Nat. Rev. Mater. 2018, 3, 113–124. 10.1038/s41578-018-0016-9.

[ref2] van den Heuvel M. G. L.; Dekker C. Motor Proteins at Work for Nanotechnology. Science 2007, 317, 333–336. 10.1126/science.1139570. 17641191

[ref3] Dreyfus R.; Baudry J.; Roper M. L.; Fermigier M.; Stone H. A.; Bibette J. Microscopic Artificial Swimmers. Nature 2005, 437, 862–865. 10.1038/nature04090. 16208366

[ref4] Vogel V. Bionic Jellyfish. Nat. Mater. 2012, 11, 841–842. 10.1038/nmat3438. 23001233

[ref5] Ricotti L.; Trimmer B.; Feinberg A. W.; Raman R.; Parker K. K.; Bashir R.; Sitti M.; Martel S.; Dario P.; Menciassi A. Biohybrid Actuators for Robotics: A Review of Devices Actuated by Living Cells. Sci. Robot. 2017, 2, eaaq0495 10.1126/scirobotics.aaq0495. 33157905

[ref6] Sengupta S.; Ibele M. E.; Sen A. Fantastic Voyage: Designing Self-Powered Nanorobots. Angew. Chem., Int. Ed. 2012, 51, 8434–8445. 10.1002/anie.201202044. 22887874

[ref7] de Ávila B. E.; Angsantikul P.; Ramírez-Herrera D. E.; Soto F.; Teymourian H.; Dehaini D.; Chen Y. J.; Zhang L. F.; Wang J. Hybrid-Biomembrane-Functionalized Nanorobots for Concurrent Removal of Pathogenic Bacteria and Toxins. Sci. Robot. 2018, 3, eaat0485 10.1126/scirobotics.aat0485. 33141704

[ref8] Wang W.; Duan W. T.; Ahmed S.; Mallouk T. E.; Sen A. Small Power: Autonomous Nano- and Micromotors Propelled by Self-Generated Gradients. Nano Today 2013, 8, 531–554. 10.1016/j.nantod.2013.08.009.

[ref9] Chen X. Z.; Jang B.; Ahmed D.; Hu C. Z.; Marco C. D.; Hoop M.; Mushtaq F.; Nelson B. J.; Pané S. Small-Scale Machines Driven by External Power Sources. Adv. Mater. 2018, 30, 1705061 10.1002/adma.201705061. 29443430

[ref10] Li J. X.; Rozen I.; Wang J. Rocket Science at the Nanoscale. ACS Nano 2016, 10, 5619–5634. 10.1021/acsnano.6b02518. 27219742

[ref11] Kim K.; Guo J. H.; Liang Z. X.; Fan D. L. Artificial Micro/Nanomachines for Bioapplications: Biochemical Delivery and Diagnostic Sensing. Adv. Funct. Mater. 2018, 28, 1705867 10.1002/adfm.201705867.

[ref12] Zhang Y. B.; Yuan K.; Zhang L. Micro/Nanomachines: From Functionalization to Sensing and Removal. Adv. Mater. Technol. 2019, 4, 1800636 10.1002/admt.201800636.

[ref13] Parmar J.; Vilela D.; Villa K.; Wang J.; Sánchez S. Micro- and Nanomotors as Active Environmental Microcleaners and Sensors. J. Am. Chem. Soc. 2018, 140, 9317–9331. 10.1021/jacs.8b05762. 29969903

[ref14] Gao W.; Wang J. The Environmental Impact of Micro/Nanomachines: A review. ACS Nano 2014, 8, 3170–3180. 10.1021/nn500077a. 24606218

[ref15] Singh V. V.; Soto F.; Kaufmann K.; Wang J. Micromotor-Based Energy Generation. Angew. Chem., Int. Ed. 2015, 54, 6896–6899. 10.1002/anie.201501971. 25906739

[ref16] de Ávila B. E.; Angsantikul P.; Li J. X.; Gao W.; Zhang L. F.; Wang J. Micromotors Go In Vivo: From Test Tubes to Live Animals. Adv. Funct. Mater. 2018, 28, 1705640 10.1002/adfm.201705640.

[ref17] Wang J. Z.; Xiong Z.; Zheng J.; Zhan X. J.; Tang J. Y. Light-Driven Micro/Nanomotor for Promising Biomedical Tools: Principle, Challenge, and Prospect. Acc. Chem. Res. 2018, 51, 1957–1965. 10.1021/acs.accounts.8b00254. 30179455

[ref18] Xu T. L.; Gao W.; Xu L. P.; Zhang X. J.; Wang S. T. Fuel-Free Synthetic Micro-/Nanomachines. Adv. Mater. 2017, 29, 1603250 10.1002/adma.201603250. 28026067

[ref19] Xu B. R.; Zhang B. R.; Wang L.; Huang G. S.; Mei Y. F. Tubular Micro/Nanomachines: From the Basics to Recent Advances. Adv. Funct. Mater. 2018, 28, 1705872 10.1002/adfm.201705872.

[ref20] Li J. X.; de Ávila B. E.; Gao W.; Zhang L. F.; Wang J. Micro/Nanorobots for Biomedicine: Delivery, Surgery, Sensing, and Detoxification. Sci. Robot. 2017, 2, eaam6431 10.1126/scirobotics.aam6431. 31552379 PMC6759331

[ref21] Wu Y. J.; Si T. Y.; Gao C. Y.; Yang M. C.; He Q. Bubble-Pair Propelled Colloidal Kayaker. J. Am. Chem. Soc. 2018, 140, 11902–11905. 10.1021/jacs.8b06646. 30176727

[ref22] Khalil I. S. M.; Magdanz V.; Sanchez S.; Schmidt O. G.; Misra S. Three-Dimensional Closed-Loop Control of Self-Propelled Microjets. Appl. Phys. Lett. 2013, 103, 172404 10.1063/1.4826141.

[ref23] Katuri J.; Ma X.; Stanton M. M.; Sánchez S. Designing Micro- and Nanoswimmers for Specific Applications. Acc. Chem. Res. 2017, 50, 2–11. 10.1021/acs.accounts.6b00386. 27809479 PMC5244436

[ref24] Eskandarloo H.; Kierulf A.; Abbaspourrad A. Light-Harvesting Synthetic Nano- and Micromotors: A review. Nanoscale 2017, 9, 12218–12230. 10.1039/c7nr05166b. 28809422

[ref25] Pourrahimi A. M.; Pumera M. Multifunctional and Self-Propelled Spherical Janus Nano/Micromotors: Recent Advances. Nanoscale 2018, 10, 16398–16415. 10.1039/C8NR05196H. 30178795

[ref26] Chen C. R.; Soto F.; Karshalev E.; Li J. X.; Wang J. Hybrid Nanovehicles: One Machine, Two Engines. Adv. Funct. Mater. 2019, 29, 1806290 10.1002/adfm.201806290.

[ref27] Ren L. Q.; Wang W.; Mallouk T. E. Two Forces Are Better than One: Combining Chemical and Acoustic Propulsion for Enhanced Micromotor Functionality. Acc. Chem. Res. 2018, 51, 1948–1956. 10.1021/acs.accounts.8b00248. 30079719

[ref28] Guix M.; Mayorga-Martinez C. C.; Merkoçi A. Nano/Micromotors in (Bio)Chemical Science Applications. Chem. Rev. 2014, 114, 6285–6322. 10.1021/cr400273r. 24827167

[ref29] Wang H.; Pumera M. Fabrication of Micro/Nanoscale Motors. Chem. Rev. 2015, 115, 8704–8735. 10.1021/acs.chemrev.5b00047. 26234432

[ref30] Ren L. Q.; Zhou D. K.; Mao Z. M.; Xu P. T.; Huang T. J.; Mallouk T. E. Rheotaxis of Bimetallic Micromotors Driven by Chemical-Acoustic Hybrid Power. ACS Nano 2017, 11, 10591–10598. 10.1021/acsnano.7b06107. 28902492

[ref31] Gao W.; Manesh K. M.; Hua J.; Sattayasamitsathit S.; Wang J. Hybrid Nanomotor: A Catalytically/Magnetically Powered Adaptive Nanowire Swimmer. Small 2011, 7, 2047–2051. 10.1002/smll.201100213. 21626685

[ref32] Chen C. R.; Tang S. S.; Teymourian H.; Karshalev E.; Zhang F. Y.; Li J. X.; Mou F. Z.; Liang Y. Y.; Guan J. G.; Wang J. Chemical/Light-Powered Hybrid Micromotors with “On-the-Fly” Optical Brakes. Angew. Chem., Int. Ed. Engl. 2018, 57, 8110–8114. 10.1002/anie.201803457. 29737003

[ref33] Hormigos R. M.; Sánchez B. J.; Escarpa A. Multi-Light-Responsive Quantum Dot Sensitized Hybrid Micromotors with Dual-Mode Propulsion. Angew. Chem., Int. Ed. Engl. 2019, 58, 3128–3132. 10.1002/anie.201811050. 30521672

[ref34] Li J. X.; Li T. L.; Xu T. L.; Kiristi M.; Liu W. J.; Wu Z. G.; Wang J. Magneto-Acoustic Hybrid Nanomotor. Nano Lett. 2015, 15, 4814–4821. 10.1021/acs.nanolett.5b01945. 26077325

[ref35] Tang S. S.; Zhang F. Y.; Zhao J.; Talaat W.; Soto F.; Karshalev E.; Chen C. R.; Hu Z. H.; Lu X. L.; Li J. X.; Lin Z. H.; Dong H. F.; Zhang X. J.; Nourhani A.; Wang J. Structure-Dependent Optical Modulation of Propulsion and Collective Behavior of Acoustic/Light-Driven Hybrid Microbowls. Adv. Funct. Mater. 2019, 29, 1809003 10.1002/adfm.201809003.

[ref36] Wu Y. J.; Wu Z. G.; Lin X. K.; He Q.; Li J. B. Autonomous Movement of Controllable Assembled Janus Capsule Motors. ACS Nano 2012, 6, 10910–10916. 10.1021/nn304335x. 23153409

[ref37] He W. P.; Frueh J.; Hu N. R. S.; Liu L. P.; Gai M. Y.; He Q. Guidable Thermophoretic Janus Micromotors Containing Gold Nanocolorifiers for Infrared Laser Assisted Tissue Welding. Adv. Sci. 2016, 3, 1600206 10.1002/advs.201600206. PMC5157175 27981009

[ref38] Šípová-Jungová H.; Andrén D.; Jones S.; Käll M. Nanoscale Inorganic Motors Driven by Light: Principles, Realizations, and Opportunities. Chem. Rev. 2020, 120, 269–287. 10.1021/acs.chemrev.9b00401. 31869216

[ref39] Hermanova S.; Pumera M. Polymer Platforms for Micro- and Nanomotor Fabrication. Nanoscale 2018, 10, 7332–7342. 10.1039/c8nr00836a. 29638234

[ref40] Meeuwissen S. A.; Kim K. T.; Chen Y. C.; Pochan D. J.; van Hest J. C. M. Controlled Shape Transformation of Polymersome Stomatocytes. Angew. Chem., Int. Ed. 2011, 50, 7070–7073. 10.1002/anie.201102167. 21688372

[ref41] Meeuwissen S. A.; Bruekers S. M. C.; Chen Y. C.; Pochan D. J.; van Hest J. C. M. Spontaneous Shape Changes in Polymersomes via Polymer/Polymer Segregation. Polym. Chem. 2014, 5, 489–501. 10.1039/C3PY00906H.

[ref42] Kim K. T.; Zhu J. H.; Meeuwissen S. A.; Cornelissen J. J. L. M.; Pochan D. J.; Nolte R. J. M.; van Hest J. C. M. Polymersome Stomatocytes: Controlled Shape Transformation in Polymer Vesicles. J. Am. Chem. Soc. 2010, 132, 12522–12524. 10.1021/ja104154t. 20718470

[ref43] Pijpers I. A. B.; Cao S. P.; Llopis-Lorente A.; Zhu J. Z.; Song S. D.; Joosten R. R. M.; Meng F. H.; Friedrich H.; Williams D. S.; Sánchez S.; van Hest J. C. M.; Abdelmohsen L. K. E. A. Hybrid Biodegradable Nanomotors through Compartmentalized Synthesis. Nano Lett. 2020, 20, 4472–4480. 10.1021/acs.nanolett.0c01268. 32427492 PMC7291354

[ref44] Wilson D. A.; Nolte R. J. M.; van Hest J. C. M. Autonomous Movement of Platinum-Loaded Stomatocytes. Nat. Chem. 2012, 4, 268–274. 10.1038/nchem.1281. 22437710

[ref45] Abdelmohsen L. K. E. A.; Nijemeisland M.; Pawar G. M.; Janssen G. A.; Nolte R. J. M.; van Hest J. C. M.; Wilson D. A. Dynamic Loading and Unloading of Proteins in Polymeric Stomatocytes: Formation of an Enzyme-Loaded Supramolecular Nanomotor. ACS Nano 2016, 10, 2652–2660. 10.1021/acsnano.5b07689. 26811982

[ref46] Nijemeisland M.; Abdelmohsen L. K. E. A.; Huck W. T. S.; Wilson D. A.; van Hest J. C. M. A Compartmentalized Out-of-Equilibrium Enzymatic Reaction Network for Sustained Autonomous Movement. ACS Cent. Sci. 2016, 2, 843–849. 10.1021/acscentsci.6b00254. 27924313 PMC5126709

[ref47] Kim K. T.; Cornelissen J. J. L. M.; Nolte R. J. M.; van Hest J. C. M. A Polymersome Nanoreactor with Controllable Permeability Induced by Stimuli-Responsive Block Copolymers. Adv. Mater. 2009, 21, 2787–2791. 10.1002/adma.200900300.

[ref48] Barreiro A.; Rurali R.; Hernández E. R.; Moser J.; Pichler T.; Forró L.; Bachtold A. Subnanometer Motion of Cargoes Driven by Thermal Gradients Along Carbon Nanotubes. Science 2008, 320, 775–778. 10.1126/science.1155559. 18403675

[ref49] Qin W. W.; Peng T. H.; Gao Y. J.; Wang F.; Hu X. C.; Wang K.; Shi J. Y.; Li D.; Ren J. C.; Fan C. H. Catalysis-Driven Self-Thermophoresis of Janus Plasmonic Nanomotors. Angew. Chem., Int. Ed. 2017, 56, 515–518. 10.1002/anie.201609121. 27921355

[ref50] Jiang H. R.; Yoshinaga N.; Sano M. Active Motion of a Janus Particle by Self-Thermophoresis in a Defocused Laser Beam. Phys. Rev. Lett. 2010, 105, 268302 10.1103/PhysRevLett.105.268302. 21231718

[ref51] Tsuji T.; Saita S.; Kawano S. Thermophoresis of a Brownian Particle Driven by Inhomogeneous Thermal Fluctuation. Phys. A 2018, 493, 467–482. 10.1016/j.physa.2017.11.145.

[ref52] Ma X.; Hahn K.; Sanchez S. Catalytic Mesoporous Janus Nanomotors for Active Cargo Delivery. J. Am. Chem. Soc. 2015, 137, 4976–4979. 10.1021/jacs.5b02700. 25844893 PMC4440854

[ref53] Dong R. F.; Zhang Q. L.; Gao W.; Pei A.; Ren B. Y. Highly Efficient Light-Driven TiO_2_-Au Janus Micromotors. ACS Nano 2016, 10, 839–844. 10.1021/acsnano.5b05940. 26592971

[ref54] Betancor L.; Hidalgo A.; Fernández-Lorente G.; Mateo C.; Fernández-Lafuente R.; Guisan J. M. Preparation of a Stable Biocatalyst of Bovine Liver Catalase Using Immobilization and Postimmobilization Techniques. Biotechnol. Prog. 2003, 19, 763–767. 10.1021/bp025785m. 12790636

[ref55] Sanchez S.; Solovev A. A.; Mei Y. F.; Schmidt O. G. Dynamic of Biocatalytic Microengines Mediated by Variable Friction Control. J. Am. Chem. Soc. 2010, 132, 13144–13145. 10.1021/ja104362r. 20860367

[ref56] Howse J. R.; Jones R. A. L.; Ryan A. J.; Gough T.; Vafabakhsh R.; Golestanian R. Self-Motile Colloid Particles: From Directed Propulsion to Random Walk. Phys. Rev. Lett. 2007, 99, 048102 10.1103/PhysRevLett.99.048102. 17678409

[ref57] Palacci J.; Sacanna S.; Steinberg A. P.; Pine D. J.; Chaikin P. M. Living Crystals of Light-Activated Colloidal Surfers. Science 2013, 339, 936–940. 10.1126/science.1230020. 23371555

[ref58] Ibele M.; Mallouk T. E.; Sen A. Schooling Behavior of Light-Powered Autonomous Micromotors in Water. Angew. Chem., Int. Ed. 2009, 48, 3308–3312. 10.1002/anie.200804704. 19338004

[ref59] Schnitzer M. J. Theory of Continuum Random Walks and Application to Chemotaxis. Phys. Rev. E 1993, 48, 2553–2568. 10.1103/physreve.48.2553. 9960890

[ref60] Shields C. W.; Velev O. D. The Evolution of Active Particles: Toward Externally Powered Self-Propelling and Self-Reconfiguring Particle Systems. Chem 2017, 3, 539–559. 10.1016/j.chempr.2017.09.006.

[ref61] Theurkauff I.; Cottin-Bizonne C.; Palacci J.; Ybert C.; Bocquet L. Dynamic Clustering in Active Colloidal Suspensions with Chemical Signaling. Phys. Rev. Lett. 2012, 108, 268303 10.1103/PhysRevLett.108.268303. 23005020

[ref62] Buttinoni I.; Bialké J.; Kümmel F.; Löwen H.; Bechinger C.; Speck T. Dynamical Clustering and Phase Separation in Suspensions of Self-Propelled Colloidal Particles. Phys. Rev. Lett. 2013, 110, 238301 10.1103/PhysRevLett.110.238301. 25167534

[ref63] Rivière D.; Selva B.; Chraibi H.; Delabre U.; Delville J. Convection Flows Driven by Laser Heating of a Liquid Layer. Phys. Rev. E 2016, 93, 023112 10.1103/PhysRevE.93.023112. 26986418

[ref64] Manna R. K.; Shklyaev O. E.; Kauffman J.; Tansi B.; Sen A.; Balazs A. C. Light-Induced Convective Segregation of Different Sized Microparticles. ACS Appl. Mater. Interfaces 2019, 11, 18004–18012. 10.1021/acsami.9b03089. 30990309

[ref65] Jin C. M.; Lee W. J.; Kim D. C.; Kang T. W.; Choi I. Photothermal Convection Lithography for Rapid and Direct Assembly of Colloidal Plasmonic Nanoparticles on Generic Substrates. Small 2018, 14, 1803055 10.1002/smll.201803055. 30294867

[ref66] Deng Z. Y.; Mou F. Z.; Tang S. W.; Xu L. L.; Luo M.; Guan J. G. Swarming and Collective Migration of Micromotors Under Near Infrared Light. Appl. Mater. Today 2018, 13, 45–53. 10.1016/j.apmt.2018.08.004.

