# Supplementary material for: Twin-Engine Janus Supramolecular Nanomotors with Counterbalanced Motion
Source: J Am Chem Soc. 2022 Jun 14;144(25):11246–52. doi: 10.1021/jacs.2c02682 (PMC9247982; doi:10.1021/jacs.2c02682)
Supplement: Supplementary file 1 — ja2c02682_si_001.pdf [file ja2c02682_si_001.pdf]

Supporting Information

Twin-Engine Janus Supramolecular Nanomotors with Counterbalanced Motion

Jingxin Shao, Shoupeng Cao, Hailong Che, Maria Teresa De Martino, Hanglong Wu, Loai K. E. A. Abdelmohsen,* and Jan C. M. van Hest*

Bio-Organic Chemistry, Institute for Complex Molecular Systems, Eindhoven University of Technology, P.O. Box 513 (STO 3.41), 5600 MB Eindhoven, The Netherlands.

E-mail: L. K. E. A. Abdelmohsen@tue.nl; J.C.M.v.Hest@tue.nl

Contents

1. Materials

2. Instruments

3. Methods

3.1 Synthesis of amphiphilic poly(ethylene glycol)-polystyrene block copolymer (PEG-*b*-PS)

3.2 Polymersomes and Janus polymersomes formation via solvent exchange method and sputter coating technique

3.3 Preparation of stomatocytes and Janus stomatocytes

3.4 Catalase encapsulation in stomatocytes and Janus stomatocytes

3.5 Enzyme loading efficiency

3.6 Enzyme activity assay

3.7 Motion analysis by CLSM

3.8 Motion analysis by NanoSight

4. Supplementary figures

Figure S1. ^{1}H-NMR spectrum and GPC traces of block copolymer

Figure S2. Morphological characterization of Janus stomatocytes by TEM

Figure S3. Size distribution of stomatocytes and Janus stomatocytes measured by DLS

Figure S4. Trajectory tracking of randomly selected Janus stomatocytes

Figure S5. Morphological characterization of catalase-loaded stomatocytes

Figure S6. Characterization of catalase encapsulation using AF4-UV-QELS

Figure S7. Movement analysis of enzyme (catalase) driven supramolecular nanomotors (motor 2: catalase stomatocytes)

Figure S8. Morphological characterization of catalase-loaded Janus stomatocytes

Figure S9. Quantification of catalase loading

Figure S10. Catalase activity

Figure S11. Tracking trajectories of catalase/Janus stomatocytes

Figure S12. Controllable "ON/OFF" motion of twin engine catalase/Janus stomatocytes

Figure S13. Tracking trajectories of catalase/Janus stomatocytes under laser programmable motion

Figure S14. Motion behaviors of non-coated stomatocytes upon laser irradiation

Figure S15. Collective behavior of catalase/Janus stomatocytes

5. References

6. Supplementary videos

Movie S1 Motion video of catalase/Janus stomatocytes in the absence of H_2O_2 fuel and NIR laser irradiation (AVI)

Movie S2 Motion video of catalase/Janus stomatocytes in a 0.75 wt. % H_2O_2 solution without NIR laser irradiation (AVI)

Movie S3 Motion of catalase/Janus stomatocytes in a 0 wt. % H_2O_2 solution under NIR laser irradiation (30.1 mW) (AVI)

Movie S4 Twin-engine mode of catalase/Janus stomatocytes (0.75 wt. % H_2O_2 solution / 30.1 mW NIR laser) (AVI)

Movie S5 Twin-engine mode of catalase/Janus stomatocytes (0.75 wt. % H_2O_2 solution / 35.7 mW NIR laser) (AVI)

Movie S6 Motion video of catalase/Janus stomatocytes upon laser irradiation (1 W) (WMV)

Movie S7 Motion video of catalase/Janus stomatocytes in a 0.15 wt. % H_2O_2 solution under laser irradiation (1 W) (WMV)

1. Materials

Poly(ethylene glycol) (PEG) 2000 was obtained from JenKen technology. Sodium nitrate ($NaNO_3$) was purchased from Merck. Dialysis Membrane MWCO 12-14000 Da from Spectra/Pro® was

used for dialysis during the shape transformation. Anisole, *N, N, N', N'', N''*-pentamethyl-diethylenetriamine (PMDETA), catalase from bovine liver lyophilized powder 2000-5000 U/mg, hydrogen peroxide solution (30 wt. % in H_2O) were purchased from Sigma-Aldrich. Styrene was distilled before polymerization to remove the inhibitor. Doxorubicin hydrochloride was purchased from Fluorochem. Protein bicinchoninic acid (BCA) protein assay kit was obtained from ThermoFisher Scientific. Tetrahydrofuran (THF) and 1,4-dioxane were obtained from Biosolve Chimie. Ultrafree-CL Centrifugal Filter (0.22 μm) was obtained from Merck. All chemicals and enzymes were used as received without further purification unless otherwise mentioned. Ultrapure Milli Q (Millipore) water (18.2 MΩ·cm) was used for all experiments in this work.

2. Instruments

Proton Nuclear magnetic resonance spectroscopy (^{1}H-NMR)

Proton nuclear magnetic resonance measurements were conducted on a Bruker 400 Ultrashield^{TM} spectrometer with $CDCl_3$ as a solvent and TMS as internal standard.

Gel permeation chromatography (GPC)

Molecular weights of the obtained block polymer were measured by using a Prominence GPC system (Shimadzu) with a PL gel 5 μm mixed D column (Polymer Laboratories) and a differential refractive index detector. THF was used as an eluent with a flow rate of 1 mL/min. Polystyrene standards in the range of 580~377400 g/mol were used for calibration.

Scanning electron microscopy (SEM)

Morphology of polymersomes, stomatocytes, and Janus stomatocytes was characterized by SEM (FEI Quanta 200 3D FEG). EDX elemental mapping analysis of Janus stomatocytes was performed by SEM (Phenom ProX).

Transmission electron microscopy (TEM)

To observe the morphology changes, TEM images were recorded with a FEI Tecnai G2 Sphere at 200 kV electron source and equipped with LaB6 filament and autoloader station.

Dynamic light scattering measurements (DLS)

DLS measurements were performed by using a Malvern Instruments Zetasizer (model Nano ZSP). Data were analyzed by Zetasizer software.

UV-*vis* spectrophotometer

BCA protein assay for catalase loading efficiency and catalase activity were characterized by UV-vis spectrophotometry (V-650, JASCO).

Confocal laser scanning microscopy (CLSM)

Motion behavior was observed and captured by using a two-photon (Chameleon Vision, Coherent, USA) excited confocal laser scanning microscope (Zeiss, LSM510 META).

Asymmetric Flow Field-Flow Fractionation-UV-QELS (AF4-UV-QELS)

AF4-UV-QELS was performed on a Wyatt Dualtec AF4 instrument connected to a Shimadzu LC-2030 Prominence-i system with Shimadzu LC-2030 autosampler. The AF4 was further connected to a Wyatt DAWN HELEOS II light scattering detector (MALS) installed at different angles (12.9 °, 20.6 °, 29.6 °, 37.4 °, 44.8 °, 53.0 °, 61.1 °, 70.1 °, 80.1 °, 90.0 °, 99.9 °, 109.9 °, 120.1 °, 130.5 °, 149.1 °, and 157.8 °) using a laser operating at 664.5 nm and a Wyatt Optilab Rex refractive index detector. Detectors were normalized using Bovine Serum Albumin (BSA). The processing and analysis of the LS data and radius of gyration (Rg) calculations were conducted on Astra 7 software. All AF4 fractionations were performed on an AF4 short channel with regenerated cellulose (RC) 10 kDa membrane (Millipore) and spacer of 350 μm. The AF4 channel was pre-washed with running solution of 5 mM $NaNO_3$.

NanoSight

Nanoparticle Tracking Analysis (NTA) was performed using a NanoSight (NS 300, Malvern Instruments) equipped with a 488 nm blue laser, an external laser source (660 nm, Ultralasers), and an Electron Multiplication Charge Coupled Device camera to track the motion of nanomotors.

3. Methods

3.1 Synthesis of amphiphilic poly(ethylene glycol)-polystyrene block copolymer (PEG-*b*-PS)

Amphiphilic block copolymer PEG_{45}-*b*-PS_{230} was synthesized using atom-transfer radical polymerization according to previously published procedures.[1-3] The obtained block copolymer was characterized by ^{1}H-NMR and GPC to measure the molecular weight and the size distribution. PEG_{45}-*b*-PS_{230} had a weight average molecular weight (M_w) of 30.4 kg/mol, a number average molecular weight of 27.4 kg/mol, and a polydispersity index (Đ) of 1.11.

3.2 Polymersomes and Janus polymersomes formation via solvent exchange method and sputter coating technique

For fabrication of polymersomes, 20 mg block copolymer PEG_{45}-*b*-PS_{230} was dissolved in 2 mL of a mixture of distilled THF/dioxane (4:1, v/v) in a 15 mL glass vial with a magnetic stirring bar. The vial was capped with a rubber septum and the solution was mixed for 30 min. Then, 2 mL Milli Q water was added via a syringe pump with a rate of 1 mL/h under vigorous stirring. During this process, the solution changed from transparent to a cloudy suspension. Janus polymersomes were prepared by using the sputter coating technique according to previously published protocols.[4-6] Briefly, a droplet of polymersome (100 μL) solution was drop-cast onto a clean silicon wafer to form a polymersome monolayer. After drying at room temperature, a turbo sputter coater (Quorum Technologies, K575X) was used to coat the upper side of the polymersomes, while the other side was protected by the

substrate. The Janus polymersomes were then re-dispersed into aqueous solution via ultrasound treatment.

3.3 Preparation of stomatocytes and Janus stomatocytes

Stomatocytes and Janus stomatocytes were prepared via a shape change process induced by dialysis. Solutions of the obtained polymersomes and Janus polymersomes were transferred into a pre-hydrated dialysis bag (12-14 kDa, 2 mL/cm) and dialyzed against Milli-Q water at room temperature. The dialysis water was replaced after 1 hour, followed by dialysis for at least 24 hours.

3.4 Catalase encapsulation in stomatocytes and Janus stomatocytes[3]

PEG_{45}-*b*-PS_{230} block copolymer (20 mg) was dissolved in 2 mL of dioxane / THF (1:4, v/v) and 3 mL water was added via a syringe pump with a rate of 1 mL/h under vigorous stirring. The obtained cloudy suspension was then transferred into a dialysis membrane (MWCO 12-14 kDa, flat width 25 mm), which was prior wetted in MilliQ water for 30 min, followed by dialysis against MilliQ-water (1000 mL) for at least 24 h. The volume of the obtained solution was concentrated to 2 mL, followed by adding 300 μL of a THF/dioxane (4:1. v/v) mixture and 2 mL of MilliQ water. To concentrate the solution, spin filtration was used to reduce the volume of the solution to 500 μL. Then, catalase (6 mg per mL) was added to the open neck stomatocytes, and the mixture was stirred for at least 30 min, followed by addition of a mixture of THF/dioxane (4:1, v/v) (150 μL, 300 μL/h) to narrow the neck of stomatocytes. To remove the organic solvent and non-loaded enzymes, stomatocytes were purified via spin filtration and dialysis using 5 mM $NaNO_3$ solution. For preparation of catalase-loaded Janus stomatocytes, the same procedure was applied with the Janus polymersomes.

3.5 Enzyme loading efficiency

The Bicinchoninic acid (BCA) protein assay was used to quantify the loading efficiency of catalase. The protein concentration of all the samples was measured according to the instruction in the user guide. These samples include polymersomes, Janus polymersomes, stomatocytes, catalase-loaded stomatocytes, Janus stomatocytes, and catalase-loaded Janus stomatocytes.

3.6 Enzyme activity assay

Enzyme activity after encapsulation was evaluated by the catalase activity assay. The UV spectrophotometer was calibrated with PBS buffer as a blank. Samples and H_2O_2 solution were added to a quartz cuvette. The final concentration of H_2O_2 was 10 mM. The cuvette was placed into the UV spectrophotometer immediately, and the decrease in the absorbance at OD_{240} was monitored. Catalase activity was then calculated according to the following equation[7]:

$$2\,\mathrm{H_2}O_2 \xrightarrow{\text{Catalase}} 2\,\mathrm{H_2O} + O_2$$

$$\mathrm{U/mg} = \frac{(A_0 - A_{60}) \times Vt}{\varepsilon_{240} \times \mathrm{d} \times \mathrm{V}_s \times \mathrm{C}_t \times 0.001}$$

Where

(A_0-A_{60})	is the difference between the initial and final absorbance.
V_t	is the total reaction volume in mL.
ε_{240}	is the molar extinction coefficient for H_2O_2 at OD_{240} (34.9 mol/cm)
d	is the optical length path of the cuvette.
V_s	is the volume of the sample in mL.
C_t	is the protein concentration of the sample in mg/mL.

3.7 Motion analysis by CLSM

To observe the motion behavior of the supramolecular nanomotors with single and dual modes, confocal laser scanning microscopy (CLSM) equipped with a two-photon laser was used. For NIR propelled nanomotors (motor 1: Janus stomatocytes), Janus- stomatocytes (30 μL, 0.4 mg/mL) were added to ibidi-8 wells which were pre-filled with 200 μL Milli-Q water. The motion of Janus-stomatocytes was recorded by scanning in the xyt mode. For enzyme-propelled nanomotors (motor 2: catalase stomatocytes), ibidi-8 wells were filled with either 0.15 wt. % H_2O_2 or 0.75 wt. % H_2O_2 solution, followed by adding 30 μL catalase stomatocytes (0.4 mg/mL). The motion frames of catalase stomatocytes were recorded by CLSM. For the motility of the twin-engines, 30 μL catalase-loaded Janus stomatocytes (motor 3: catalase/Janus stomatocytes) were added to the corresponding H_2O_2 solution (0.15 wt. % or 0.75 wt. %). The motion frames of catalase/Janus stomatocytes were recorded by conducting xyz scanning under NIR irradiation (760 nm) at different laser intensities. All the experiments were performed at room temperature.

To analyze motion, the corresponding videos of each group were exported by the CLSM software (ZEN 2012, Zeiss). The motion coordinate information of each nanomotor was obtained by manual tracking via ImageJ, thus providing the tracking trajectory of the nanomotors. Based on the tracking trajectory, the total pathway of the nanomotors could be obtained, thus the corresponding velocity was calculated by dividing the total pathway with the time. The mean square displacement (MSD) was analyzed according to a previously published protocol. For the case of two-dimensional analysis, the MSD was calculated following this formula,[8]

$$\text{MSD} = (x(\Delta t) - x(0))^2 + (y(\Delta t) - y(0))^2$$

The tracking trajectory, MSD, and velocity of the supramolecular nanomotors were calculated from 20 randomly selected motors.

The swarming behavior of catalase/Janus stomatocytes (motor 3) was investigated by analyzing the change in fluorescence intensity as a function of time using ImageJ.

3.8 Motion analysis by NanoSight

To investigate the autonomous motion of Janus stomatocytes, catalase stomatocytes, and catalase/Janus stomatocytes, nanoparticle tracking analysis was also performed using NanoSight equipped with an external laser source (660 nm). For a typical experiment, 1 mL of diluted sample was loaded in the NTA chamber using a plastic syringe (1 mL, without needle). The approximate concentration of Janus stomatocytes, catalase stomatocytes, and catalase/Janus stomatocytes were 2.08 $\times 10^8$, 3.23 $\times 10^8$, and 7.51 $\times 10^8$ particles/mL respectively. The NTA 2.2 software allowed to extract and track the trajectory of single nanoparticles. The motion of nanomotors was recorded by the NTA for 30 seconds. To study the enzyme-driven mode, samples were mixed with an aliquot of hydrogen peroxide. The measurements were started immediately after mixing and injecting the samples (catalase stomatocytes, and catalase/Janus stomatocytes) to the NTA chamber.

The NTA technique allows the two-dimensional extraction of coordinates (x,y) by recording the trajectories of the nanoparticles. Hence, we measured the motion of each group by tracking at least 30 nanoparticles for 30 seconds and presented the motion pathway of each nanomotor. The MSD of each nanomotor was calculated using the followed equation,[9]

$$\mathrm{MSD} = (4\mathrm{D})\Delta \mathrm{t} + (V^2)(\Delta t^2)$$

From the MSD fitting curves, the movement behavior of the nanomotors could be determined. According to the Golestanian's self-diffusiophoretic model,[10] a linear MSD curve can be obtained if the nanomotors are in Brownian motion. At this situation, the equation used for extracting MSD should be $\mathrm{MSD} = (4\mathrm{D})\Delta \mathrm{t}$.[9] Self-propelled motion should result in a nonlinear MSD fitting curve. By correlating laser power and fuel concentration (H_2O_2 concentration) to the MSD curve, it was obvious that a higher laser power or fuel concentration led to a transition of the MSD curve from a linear to a parabolic fit. The average velocity of each group was extracted from the fitting of the average MSD curves, according to the previously published method.[11, 12]

4. Supplementary figures

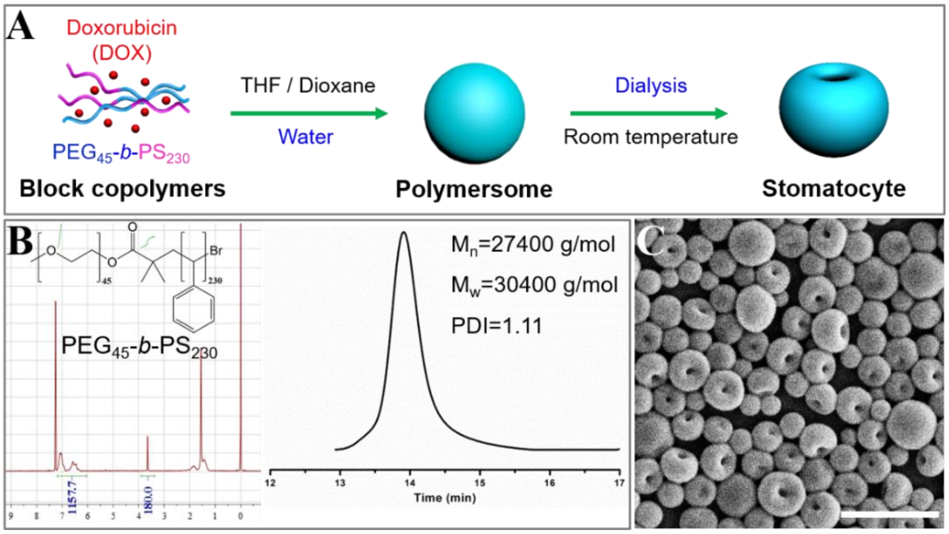

Figure S1. (A) Schematic illustration of preparation of stomatocytes. (B) ^{1}H-NMR spectrum (left) and GPC traces (right) of PEG$_{45}$-*b*-PS$_{230}$. (C) SEM image, scale bar = 500 nm.

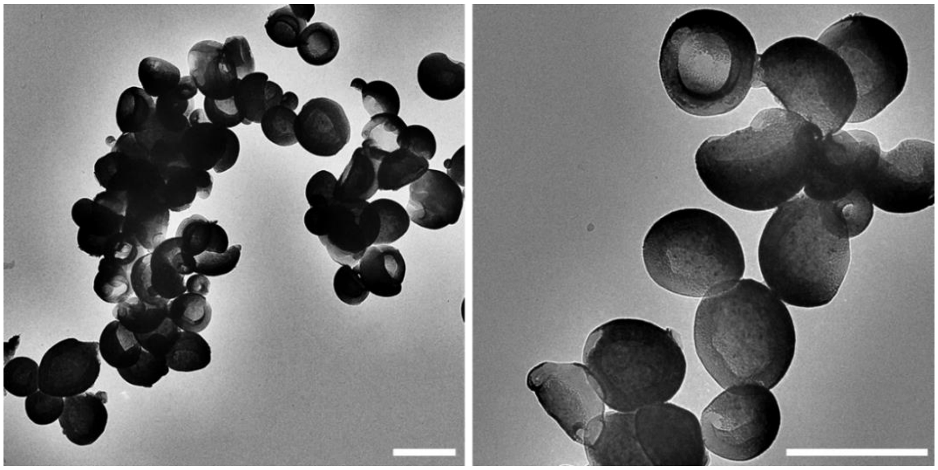

Figure S2. Morphological characterization of Janus stomatocytes by TEM. Scale bar = 500 nm.

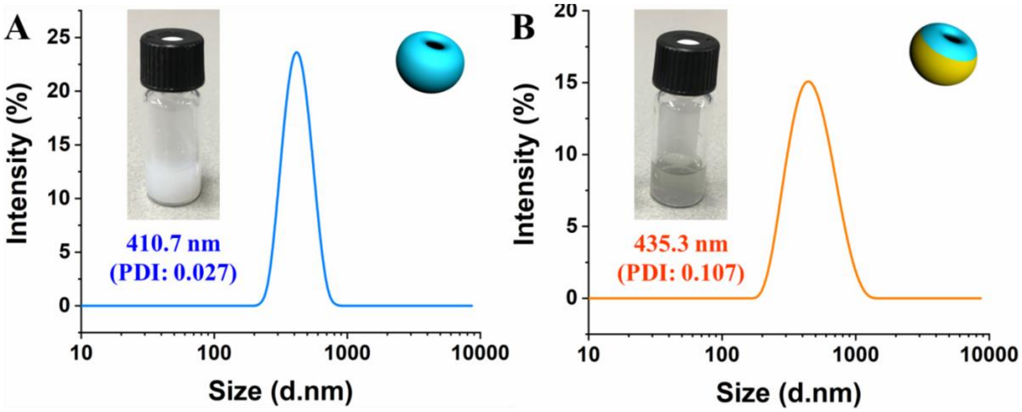

Figure S3. Size distribution of stomatocytes (A) and Janus stomatocytes (B) measured by dynamic light scattering. The Insert image is the optical image of stomatocytes and Janus stomatocytes. After gold coating, the color of the stomatocytes solution was changed from cloudy white to cyan.

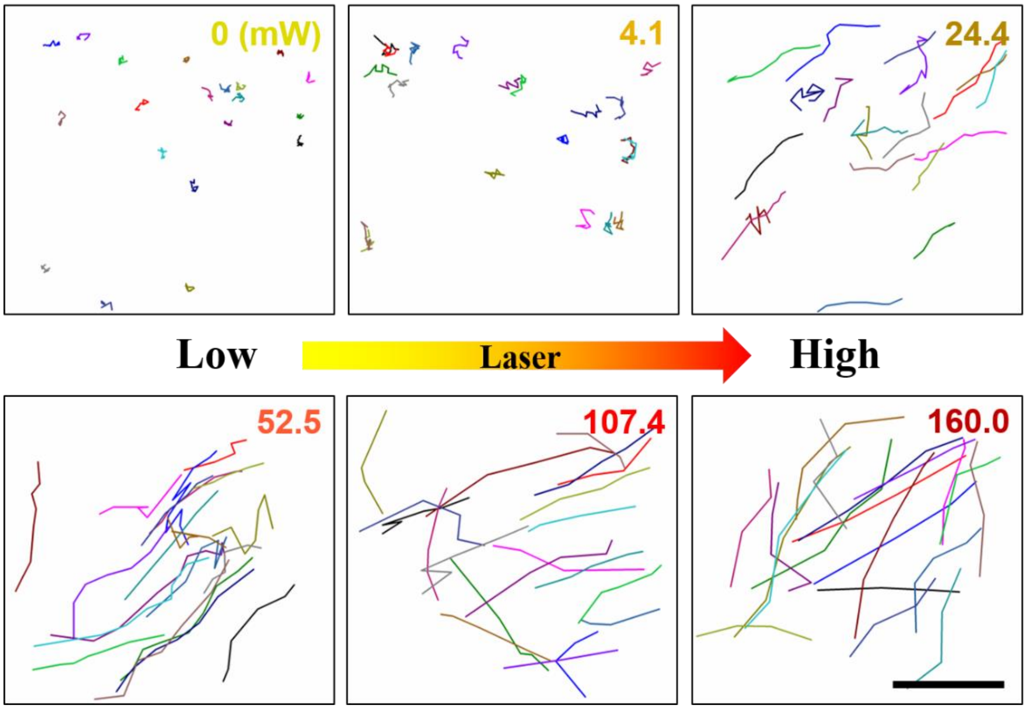

Figure S4. Trajectory tracking of randomly selected Janus stomatocytes as a function of output laser power, scale bar = 50 μm.

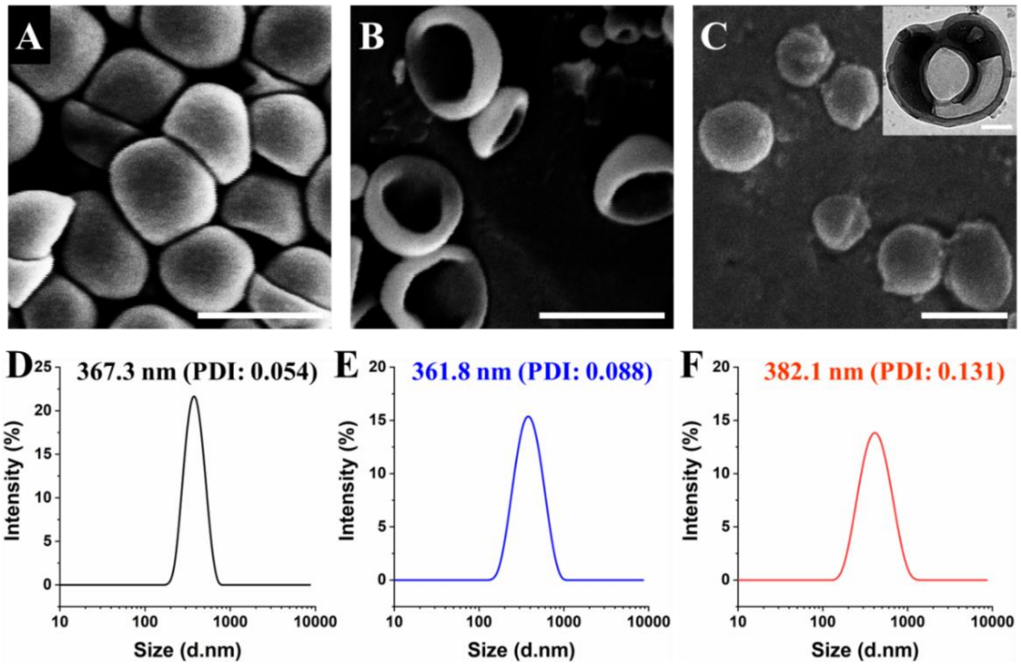

Figure S5. Morphological characterization during the preparation of catalase-loaded stomatocytes. (A) SEM images of polymersomes with glassy membrane; (B) wide open neck stomatocytes and (C) narrow neck stomatocytes after loading with catalase. Scale bar = 500 nm. Insert image in C is the TEM image of a narrow neck stomatocyte, scale bar = 100 nm. Size distribution of polymersomes with glassy membrane (D), wide open neck stomatocytes (E), and narrow neck stomatocytes after loading with catalase (F).

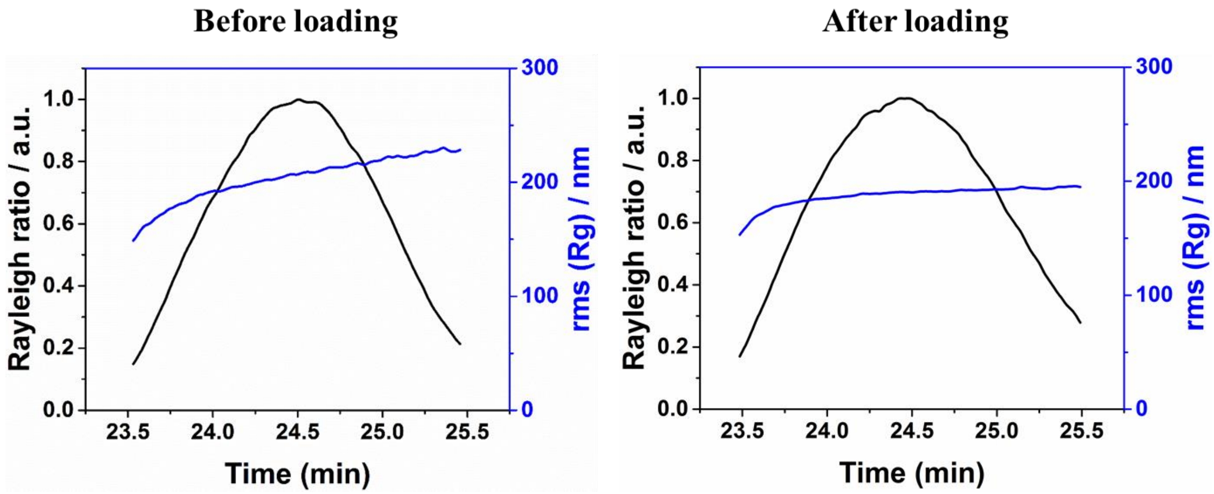

Figure S6. Characterization of catalase encapsulation. AF4-UV-QELS of empty stomatocytes (left) and catalase loaded stomatocytes purified by spin filtration (right).

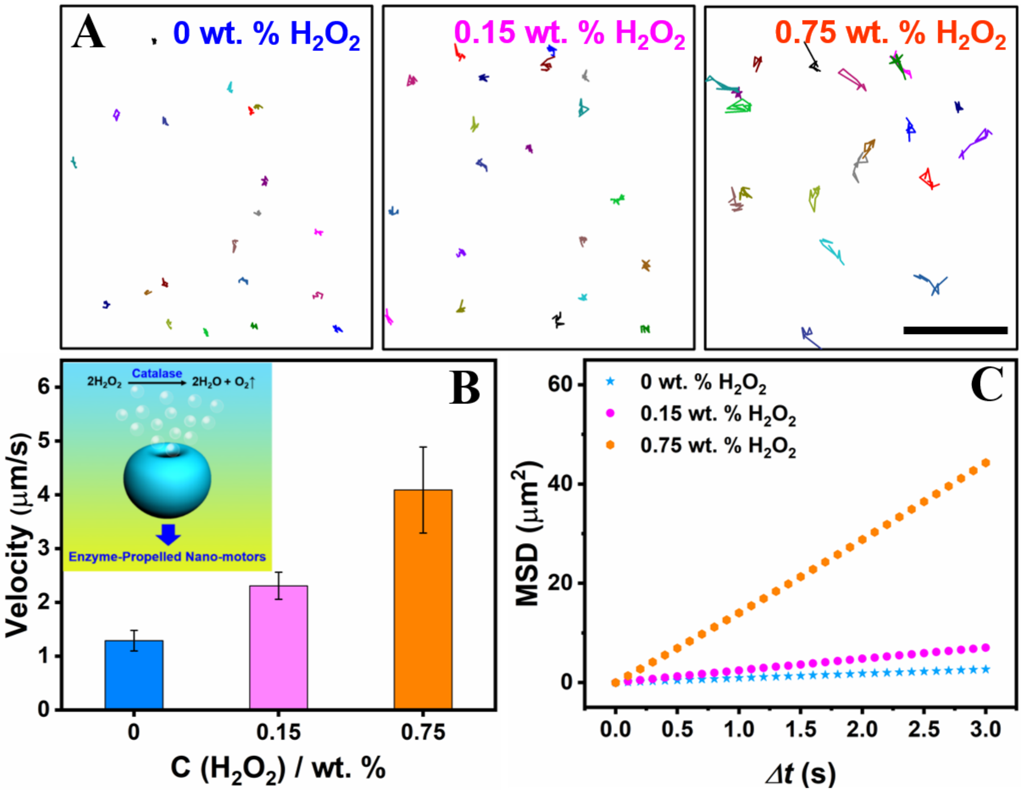

Figure S7. Movement analysis of enzyme (catalase) driven supramolecular nanomotors (motor 2: catalase stomatocytes). (A) Tracking lines of randomly selected stomatocyte nanomotors in the presence of H_2O_2 with different concentration, scale bar = 50 μm. (B) Velocity distribution of stomatocyte nanomotors as a function of H_2O_2 concentration. (C) MSD versus time interval (Δt) analyzed from tracking trajectories.

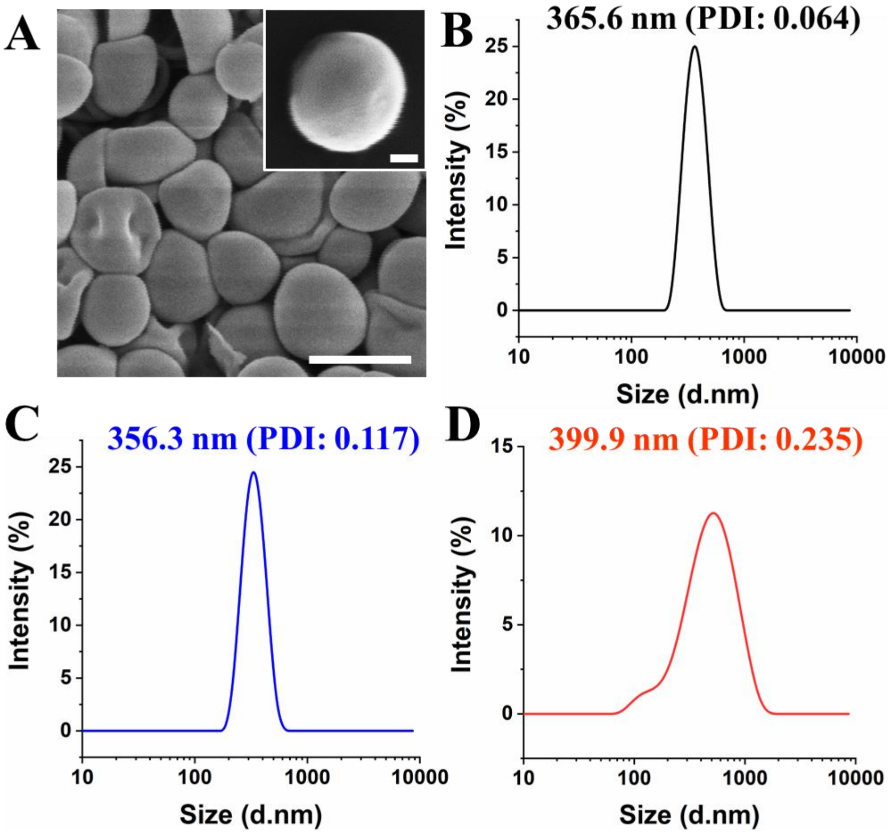

Figure S8. (A) SEM images of Janus polymersomes, scale bar = 500 nm. Insert image is the corresponding SEM image with higher magnification, scale bar = 100 nm. Size distribution of Janus polymersomes (B), wide open neck Janus stomatocytes (C), and narrow neck Janus stomatocytes after loading with catalase (D).

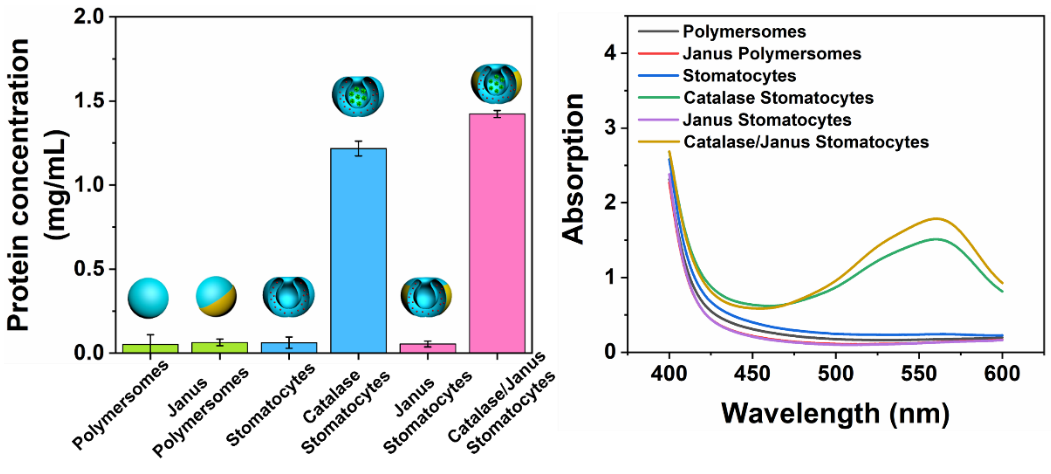

Figure S9. Quantification of catalase loading by using a BCA protein assay (left) and the corresponding UV-vis absorption spectra (right).

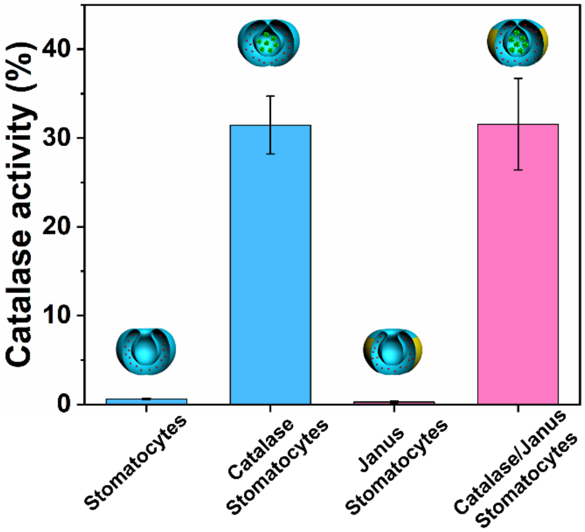

Figure S10. Catalase activity of stomatocytes, catalase stomatocytes, Janus stomatocytes, and catalase/Janus stomatocytes.

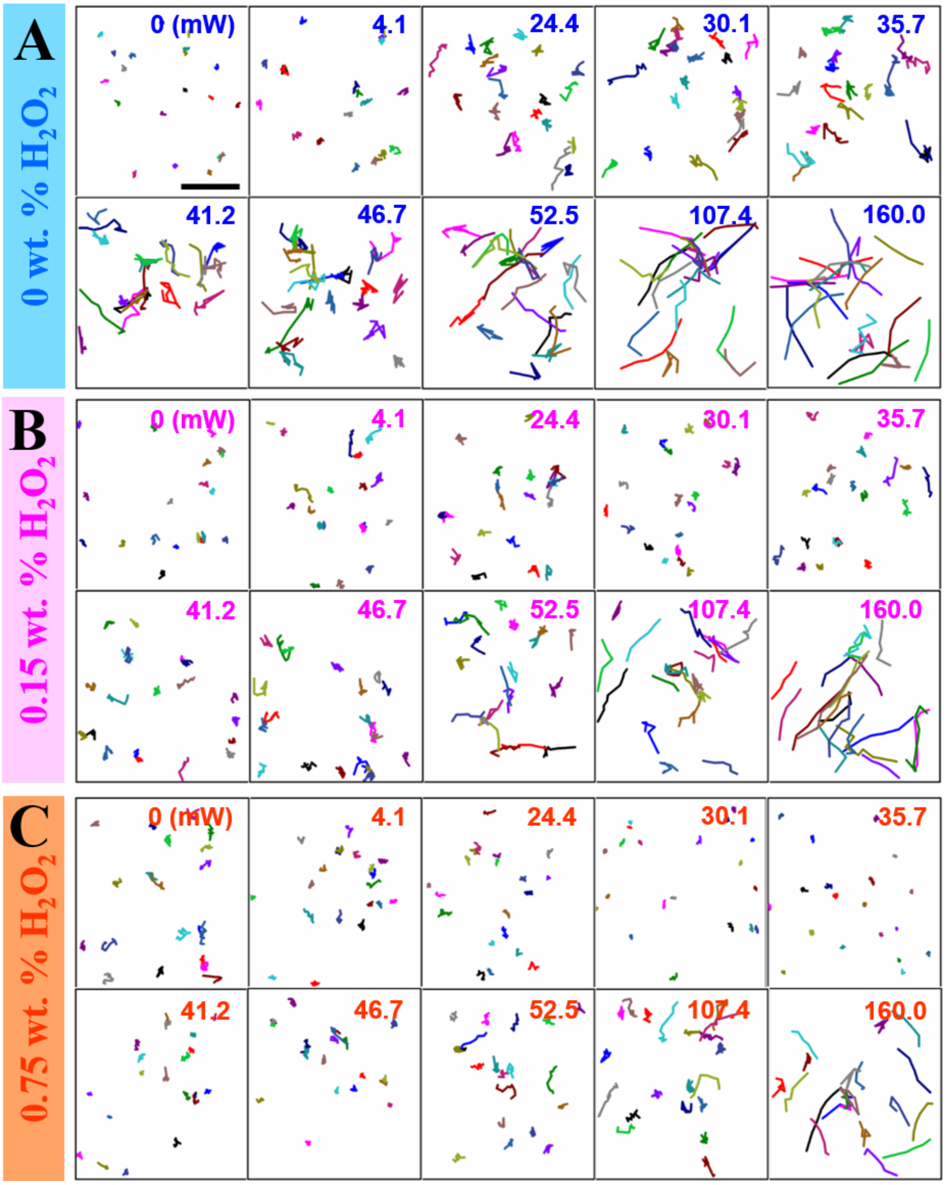

Figure S11. Tracking trajectories of catalase/Janus stomatocytes as a function of incident laser power with different H_2O_2 concentrations, including 0 wt. % H_2O_2 (A), 0.15 wt. % H_2O_2 (B), and 0.75 wt. % H_2O_2 (C). Scale bar = 50 μm.

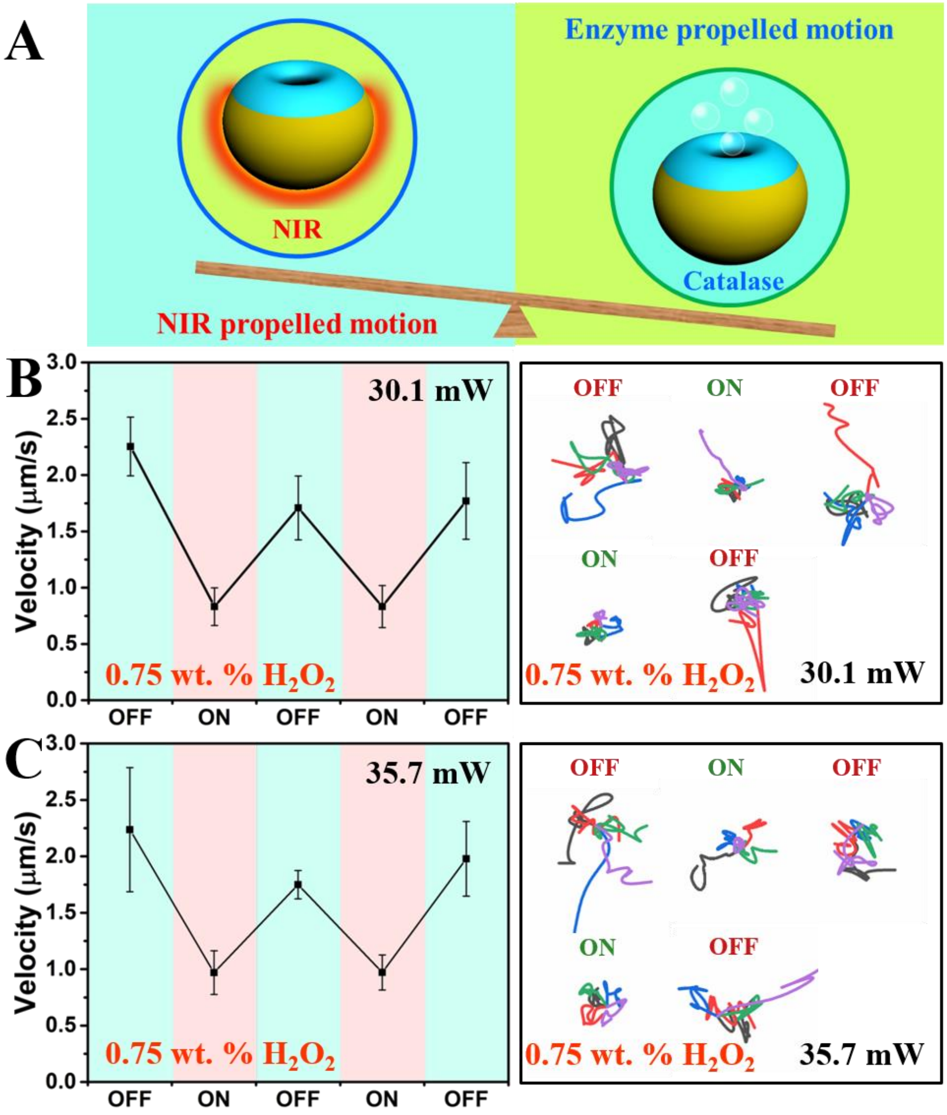

Figure S12. Controllable "ON/OFF" motion of twin-engine catalase/Janus stomatocytes. (A) Schematic illustration of the "seesaw effect" induced by the opposite movement direction of catalase/Janus stomatocytes. Under NIR irradiation, nanomotors display a NIR-driven mode and move with the nanocavity to the front (left). Nanomotors switch to enzyme-driven mode in the presence of H_2O_2 (right). Catalase encapsulated into the stomatocytes catalytically decomposes H_2O_2 to generate oxygen, which in turn pushes the nanomotors forward, now with the cavity pointing to the back towards the gold side. (B) Velocity of "ON/OFF" motion of catalase/Janus stomatocytes in the presence of 0.75 wt. % H_2O_2 with laser irradiation (30.1 mW) and tracking trajectories. (C) "ON/OFF" motion of catalase/Janus stomatocytes in presence of 0.75 wt. % H_2O_2 and irradiated with 35.7 mW NIR laser.

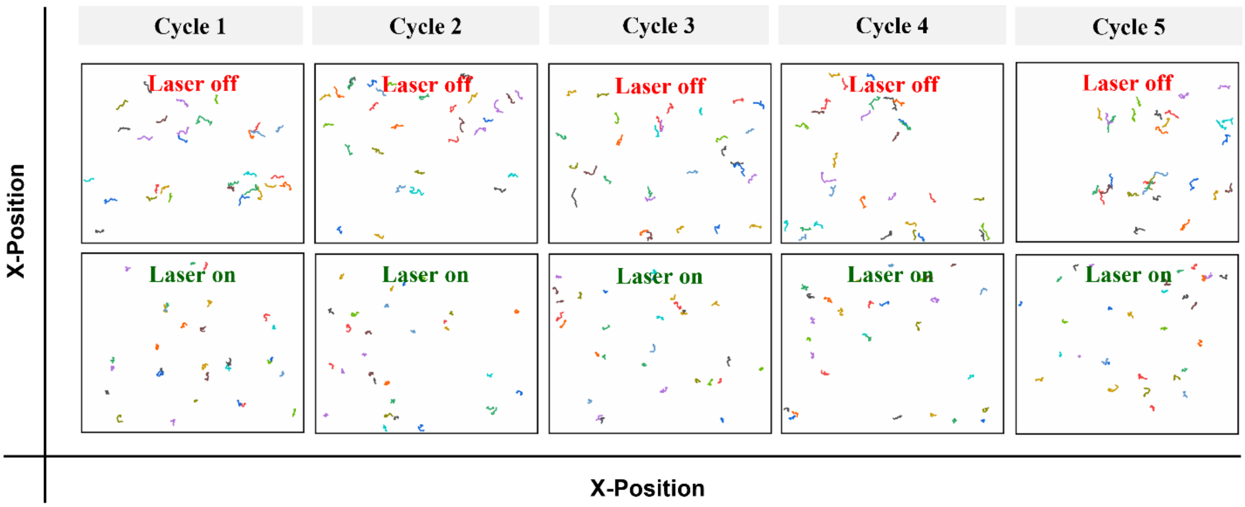

Figure S13. Tracked trajectory of catalase/Janus stomatocytes under laser programmable motion.

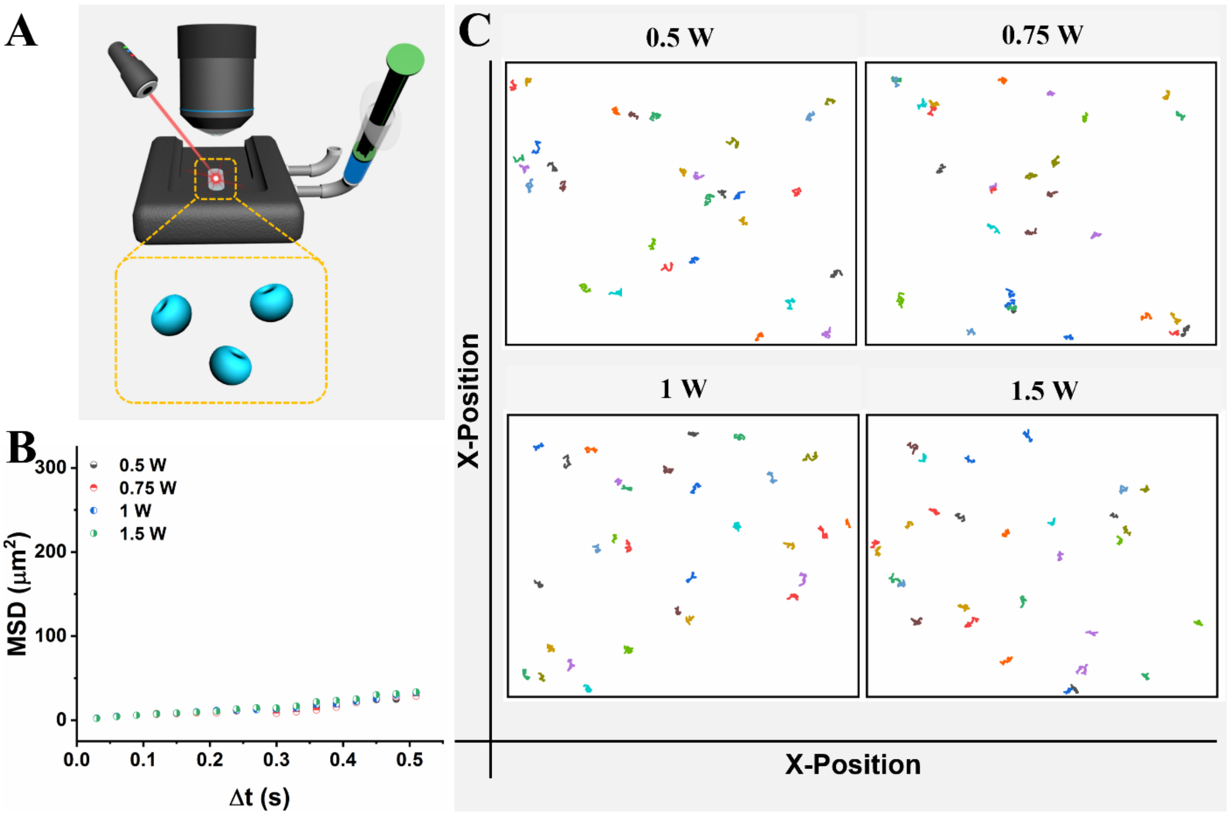

Figure S14. Motion behaviors of uncoated stomatocytes upon laser irradiation. (A) Schematic illustration of the characterization of motion using nanoparticle tracking analysis (NTA) technique. (B) MSD of uncoated stomatocytes as a function of output laser power. (C) Corresponding motion trajectories of uncoated stomatocytes under laser irradiation.

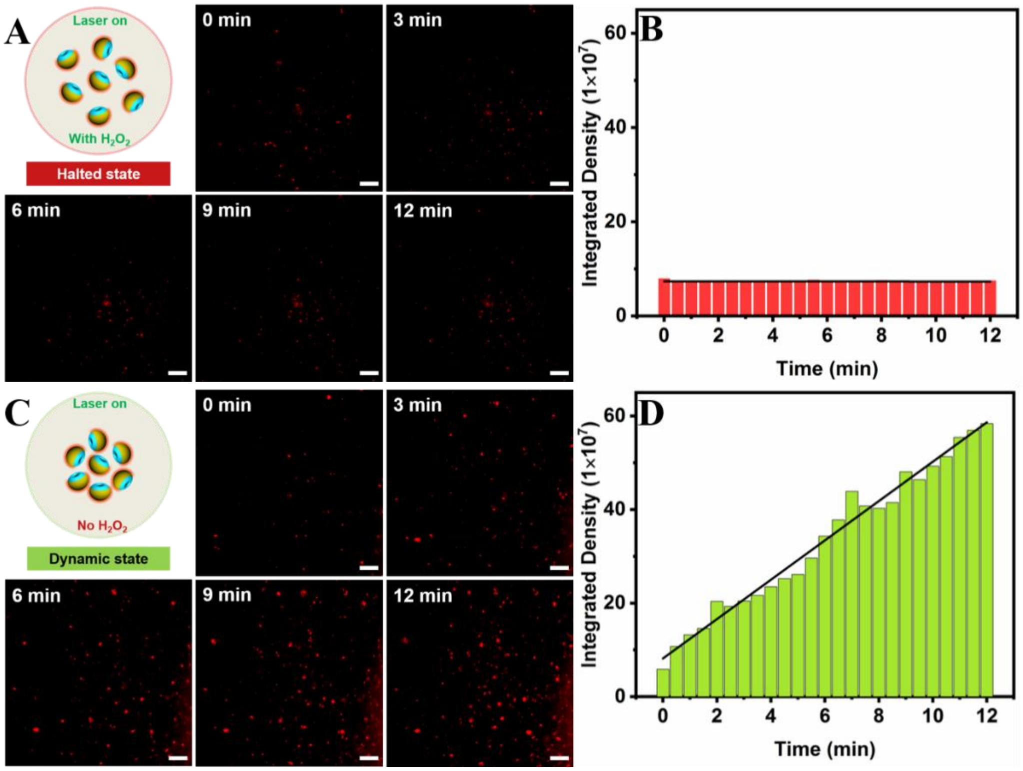

Figure S15. Collective behavior of catalase/Janus stomatocytes, modulated by tuning the net outcome of the two co-existing driving forces. (A) Time-lapsed CLSM images of the halted state. The catalase/Janus stomatocytes were powered with both NIR laser light and H_2O_2. Scale bar = 20 μm. (B) The integrated density of CLSM images as a function of time analysis by ImageJ. (C) Time-lapsed images of the catalase/Janus stomatocytes in dynamic mode. The main driving force here was provided by NIR laser light. Scale bar = 20 μm. (D) The fluorescence intensity of the time lapsed CLSM images.

5. References

1. Kim, K. T.; Cornelissen, J. J. L. M.; Nolte, R. J. M.; van Hest, J. C. M. A Polymersome Nanoreactor with Controllable Permeability Induced by Stimuli-Responsive Block Copolymers. *Adv. Mater.* **2009**, *21*, 2787-2791.

2. Kim, K. T.; Zhu, J. H.; Meeuwissen, S. A.; Cornelissen, J. J. L. M.; Pochan, D. J.; Nolte, R. J. M.; van Hest, J. C. M. Polymersome Stomatocytes: Controlled Shape Transformation in Polymer Vesicles. *J. Am. Chem. Soc.* **2010**, *132*, 12522-12524.

3. Abdelmohsen, L. K. E. A.; Nijemeisland, M.; Pawar, G. M.; Janssen, G. A.; Nolte, R. J. M.; van Hest, J. C. M.; Wilson, D. A. Dynamic Loading and Unloading of Proteins in Polymeric Stomatocytes: Formation of an Enzyme-Loaded Supramolecular Nanomotor. *ACS Nano* **2016**, *10*, 2652-2660.

4. Wu, Y. J.; Si, T. Y.; Shao, J. X.; Wu, Z. G.; He, Q. Near-Infrared Light-Driven Janus Capsule Motors: Fabrication, Propulsion, and Simulation. *Nano Res.* **2016**, *9*, 3747-3756.

5. Dong, R. F.; Hu, Y.; Wu, Y. F.; Gao, W.; Ren, B. Y.; Wang, Q. L.; Cai, Y. P. Visible-Light-Driven BiOI-Based Janus Micromotor in Pure Water. *J. Am. Chem. Soc.* **2017**, *139*, 1722-1725.

6. Zong, Y. W.; Liu, J.; Liu, R.; Guo, H. L.; Yang, M. C.; Li, Z. Y.; Chen, K. An Optically Driven Bistable Janus Rotor with Patterned Metal Coatings. *ACS Nano* **2015**, *9*, 10844-10851.

7. Orta-Zavalza, E.; Briones-Martin-del-Campo, M.; Castano, I.; Penas, A. D. L. Catalase Activity Assay in Candida glabrata. *Bio-Protocol* **2014**, *4*, e1072.

8. Ma, X.; Hahn, K.; Sanchez, S. Catalytic Mesoporous Janus Nanomotors for Active Cargo Delivery. *J. Am. Chem. Soc.* **2015**, *137*, 4976-4979.

9. Che, H. L.; Zhu, J. Z.; Song, S. D.; Mason, A. F.; Cao, S. P.; Pijpers, I. A. B.; Abdelmohsen, L. K. E. A.; van Hest, J. C. M. ATP-Mediated Transient Behavior of Stomatocyte Nanosystems. *Angew. Chem. Int. Ed.* **2019**, *58*, 13113-13118.

10. Howse, J. R.; Jones, R. A. L.; Ryan, A. J.; Gough, T.; Vafabakhsh, R.; Golestanian, R. Self-Motile Colloidal Particles: From Directed Propulsion to Random Walk. *Phys. Rev. Lett.* **2007**, *99*, 048102.

11. Nijemeisland, M.; Abdelmohsen, L. K. E. A.; Huck, W. T. S.; Wilson, D. A.; van Hest, J. C. M. A Compartmentalized Out-of-Equilibrium Enzymatic Reaction Network for Sustained Autonomous Movement. *ACS Cent. Sci.* **2016**, *2*, 843-849.

12. Pijpers, I. A. B.; Cao, S. P.; Llopis-Lorente, A.; Zhu, J. Z.; Song, S. D.; Joosten, R. R. M.; Meng, F. H.; Friedrich, H.; Williams, D. S.; Sánchez, S.; van Hest, J. C. M.; Abdelmohsen, L. K. E. A. Hybrid Biodegradable Nanomotors through Compartmentalized Synthesis. *Nano Lett.* **2020**, *20*, 4472-4480.
